# Influence of expression and purification protocols on Gα biochemical activity: kinetics of plant and mammalian G protein cycles

**DOI:** 10.3389/fmolb.2025.1513660

**Published:** 2025-04-07

**Authors:** Timothy E. Gookin, David Chakravorty, Sarah M. Assmann

**Affiliations:** Biology Department, Pennsylvania State University, University Park, PA, United States

**Keywords:** heterotrimeric G protein, recombinant protein expression, GPA1, GNAO1, GNAI1, GTP, BODIPY, SYPRO orange

## Abstract

Heterotrimeric G proteins, composed of Gα, Gβ, and Gγ subunits, are a class of signal transduction complexes with broad roles in human health and agriculturally relevant plant physiological and developmental traits. In the classic paradigm, guanine nucleotide binding to the Gα subunit regulates the activation status of the complex. We sought to develop improved methods for heterologous expression and rapid purification of Gα subunits, initially targeting GPA1, the sole canonical Gα subunit of the model plant species, *Arabidopsis thaliana.* Compared to conventional methods, our expression methodology and rapid StrepII-tag mediated purification facilitates substantially higher yield, and isolation of protein with increased GTP binding and hydrolysis activities. Human GNAI1 purified using our approach displayed the expected binding and hydrolysis activities, indicating our protocol is applicable to mammalian Gα subunits, potentially including those for which purification of enzymatically active protein has been historically problematic. We subsequently utilized domain swaps of GPA1 and human GNAO1 to demonstrate that the inherent instability of GPA1 is a function of the interaction between the Ras and helical domains. Additionally, we found that GPA1-GNAO1 domain swaps partially uncouple the instability from the rapid nucleotide binding kinetics displayed by GPA1. In summary, our work provides insights into methods to optimally study heterotrimeric G proteins, and reveals roles of the helical domain in Gα kinetics and stability.

## 1 Introduction

The heterotrimeric G protein complex consists of an alpha (Gα), beta (Gβ), and gamma (Gγ) subunit, in which Gβ and Gγ exist as a non-dissociable dimer. Heterotrimeric G proteins (G proteins) are well-studied conserved eukaryotic signal transduction components. Mutations of G protein subunits in humans have been associated with diseases and developmental abnormalities including cancer (Van Raamsdonk et al., 2009; O’Hayre et al., 2013; Hewitt et al., 2023), neurodevelopmental disorders (Muir et al., 2021), McCune-Albright Syndrome (Weinstein et al., 1991), diabetes (Gawler et al., 1987; Moxham and Malbon, 1996), hypertension (Siffert et al., 1998) and ventricular tachycardia (Lerman et al., 1998). In plants, null mutants of G protein subunits have been utilized to implicate G proteins in agronomically important traits such as morphological development (Ashikari et al., 1999; Ullah et al., 2001), grain shape and yield (Botella, 2012), hormone sensitivity (Ullah et al., 2002; Trusov et al., 2007), stomatal responses (Wang et al., 2001; Coursol et al., 2003; Yu et al., 2018), salinity tolerance (Colaneri et al., 2014), drought tolerance (Ferrero-Serrano and Assmann, 2016) and pathogen resistance (Llorente et al., 2005; Trusov et al., 2006).

The Gα subunit of the G protein heterotrimer binds guanine nucleotides in a binding pocket located within a cleft between the Ras-like and helical domains of the protein. The identity of the nucleotide, GDP or GTP, determines the activation state of the heterotrimer in the canonical signaling paradigm. In the inactive heterotrimer, Gα exists in the GDP-bound form, while exchange of GDP for GTP results in activation of the heterotrimer and dissociation of Gα from the Gβγ dimer. Gα and Gβγ are then able to signal to downstream effectors, until the intrinsic GTPase activity of the Gα subunit hydrolyzes GTP to GDP, thereby stimulating reassociation of the inactive heterotrimer. The activation status of the complex can be regulated by guanine nucleotide exchange factors (GEFs) including 7-transmembrane spanning G protein-coupled receptors (GPCRs) that stimulate GTP-binding, and GTPase activating proteins (GAPs) such as regulator of G protein signaling (RGS) proteins that stimulate GTP hydrolysis (McCudden et al., 2005). In mammals the GPCR superfamily is large and perceives diverse ligands, with over 800 GPCRs encoded in the human genome (Hauser et al., 2017). In contrast, only a few 7TM proteins have been identified as candidate GPCRs in plants (Gookin et al., 2008; McFarlane et al., 2021; Gotkhindikar et al., 2025), including in the intensively studied model dicot *Arabidopsis thaliana* (Urano and Jones, 2013). Receptor-like kinases (RLKs) may predominate in the GPCR role in plants (Bommert et al., 2013; Liu et al., 2013; Ishida et al., 2014; Aranda-Sicilia et al., 2015; Chakravorty and Assmann, 2018; Yu et al., 2018).

Purification of active recombinant heterotrimeric G protein Gα subunits is integral to understanding structure-function relationships. Purification of full-length functional Gα subunits from *E. coli* has presented challenges for some Gα family members; for example, Gα_t_ consistently aggregates within inclusion bodies (Skiba et al., 1996). Previously employed strategies for recombinant Gα expression have included N-terminal protein deletions (Jones et al., 2011a; Lyon et al., 2013), chimeric substitutions (Slep et al., 2001; Chen et al., 2005; Kreutz et al., 2006) and use of insect cell expression systems (Draper-Joyce et al., 2018; Maeda et al., 2019). Here, we sought to assess the enzyme kinetics of the sole canonical *Arabidopsis* Gα subunit, GPA1, in comparison with two closely related mammalian G proteins in the Gαi family, GNAI1 and GNAO1. In the course of our investigations we addressed some of the challenges to purifying Gα proteins, finding that specific purification protocols markedly affect the activity of both plant and mammalian Gα proteins.

GPA1 has previously been described to: 1) display self-activating properties due to spontaneous nucleotide exchange and fast GTP-binding, and 2) exhibit slow GTPase activity, which skews the protein to the GTP-bound form, especially when compared to the human Gα_i1_ protein, GNAI1 (Jones et al., 2011a; Urano et al., 2012). Jones et al. (2011a) determined the structure of GPA1 by x-ray crystallography, discovering that the GPA1 tertiary structure bears a strong resemblance to that of GNAI1. Yet, GPA1 and GNAI1 also display distinctly different enzymatic activities, indicating that differences arise on a finer scale, possibly due to high levels of intrinsic disorder within, and dynamic motion of, the GPA1 helical domain (Jones et al., 2011a). To further investigate Gα enzymatic properties, we developed a robust expression and rapid purification protocol utilizing dual StrepII-tags (Schmidt and Skerra, 2007), which allowed Gα elution in a buffer directly compatible with downstream nucleotide binding and GTP hydrolysis assays, thereby abrogating any need for protracted buffer component removal, e.g., by dialysis. GPA1 exhibits increased activity when rapidly purified using our specific purification protocol. Furthermore, assays of 2 Gα subunits from the most closely related human Gα family, Gα_i_, demonstrate that mammalian Gα activity is also impacted by the choice of purification regime, and that our StrepII-tag approach to expression and purification is applicable to mammalian Gα subunits. Our results also imply that the StrepII-tag approach may represent an improved protocol for purification of other classes of enzymes including but not limited to small G proteins and other nucleotidases, due to the direct compatibility of the elution buffer with downstream applications.

## 2 Materials and methods

### 2.1 Cloning

*GPA1* was amplified from *Arabidopsis* cDNA with flanking NcoI and BspEI restriction sites. *GNAI1* with the same flanking restriction sites was amplified from a wild type clone (Genscript, clone OHu13586) and from a designed codon harmonized (Angov et al., 2008) gBlock synthesized by Integrated DNA Technologies. These Gα subunits were cloned into the NcoI and BspEI sites of pSTTa, a vector we adapted from pGEX to include N-terminal dual-StrepII tags, thrombin and TEV protease sites, a multiple cloning site and an optional C-terminal FLAG tag. *GNAO1* was amplified from a commercial clone (Genscript, clone OHu15183), adapting a 5′ BspHI restriction site (yields a sticky end compatible with NcoI) and a blunt 3′ end to clone into NcoI/PmlI sites of pSTTa. The C-terminal RGS box of *RGS1* (Chen et al., 2003), corresponding to residues 247-459, was amplified from *Arabidopsis* cDNA with flanking NcoI and BspEI sites to clone into pSTTa in the same manner as *GPA1* and *GNAI1*. All cDNAs cloned into pSTTa included a stop codon, so the open reading frame (ORF) did not read through to the C-terminal FLAG tag included in the vector. Mutants of *GPA1*, *GNAI1* and *GNAO1* were generated by REPLACR mutagenesis (Trehan et al., 2016). *GPA1-GNAO1* helical domain swaps were generated by overlap-extension PCR (Ling and Robinson, 1997) and cloned into pSTTa as above, with the exception that the *GPA1* ^*GNAO1hel*^ construct was amplified with a 5′ BspHI site. Helical domains were defined as GPA1 residues E68-Y188 and GNAO1 residues G63-R177, with the flanking sequences, GPA1 residues M1-D67 and A189-L383 or GNAO1 residues M1-S62 and T178-Y354, defined as the Ras domains, consistent with the regions used in the GPA1-GNAI1 domain swap performed by Jones et al. (2011a). His- and GST-tagged constructs were generated by amplifying the ORFs of *GPA1*, *GNAI1* and *GNAO1*, which were A-tailed, TOPO cloned into pCR8 and mobilized by LR Gateway recombination into pDEST17 (for His-tagged expression), and in the case of *GPA1*, pDEST15 (for GST-tagged expression comparisons) (Thermo Fisher Scientific). Primers for ORF cloning, mutagenesis and overlap-extension PCRs are listed in Supplementary Table S1. All sequences were verified by Sanger sequencing.

### 2.2 Protein expression

Proteins were heterologously expressed in *E. coli* BL21 DE3 cells using 75 μg/mL carbenicillin for plasmid selection. Typically, fresh transformants were grown in 7.5 mL overnight cultures (LB media supplemented with 0.5% D-glucose (w/v) and 3 g/L MgCl_2_), pelleted by centrifugation at 5,000 g for 10 min, resuspended in 5 mL fresh pre-warmed LB, and grown at 37°C. Five ml of pre-warmed HSLB (LB media supplemented with 17 g/L NaCl and 3 g/L MgCl_2_, pH 7.0) was added at T = 20 and 40 min. At T = 60 min the pre-culture was added to 600 mL prewarmed HSLB in a vigorously shaking (225 rpm) 2 L baffled flask (OD_600_ = 0.04–0.06). Cultures were grown to an OD_600_ of 0.7–0.8, transferred to a room temperature (20°C–21°C) shaker and grown for 20 min before induction with 125 μM IPTG for 3–4 h. Cells were pelleted by 6,000 g centrifugation for 10 min at 4°C. Cell pellets were promptly frozen at −80°C and typically processed the following morning, though proteins retained activity when cell pellets were stored for multiple weeks at −80°C, allowing for stockpiling of cell pellets for future processing.

### 2.3 Protein purification

All buffers were prepared with high purity premium grade reagents (e.g., Honeywell TraceSelect, Millipore Sigma BioXtra or EMD Millipore EmSure) to minimize introduction of extraneous metals, and supplemented with one tablet Complete EDTA-free protease inhibitor (Roche, 5056489001) or Pierce Protease Inhibitor Tablets, EDTA-free (Thermo Fisher Scientific, A32965) per 50 mL. Columns were pre-rinsed with 1 mL of 0.25% Tween-20. Frozen cell pellets containing expressed StrepII-tag fusion proteins were resuspended with a 10 mL Pasteur pipet in 10 mL buffer W1 (100 mM Tris-HCl, 500 mM NaCl, 2 mM MgCl_2_, 5 mM TCEP and 5% glycerol pH 8.0) supplemented with ∼10 mg lysozyme (Millipore Sigma, L1667), 25 μL/mL BioLock biotin blocker (IBA, 2-0205-050) and 5 μL Pierce Universal Nuclease (Thermo Fisher Scientific, 88702), and kept on ice. Cells were lysed by three rounds of sonication on ice using a Fisher Sonic Dismembrator equipped with a 3 mm tip with 1 s on/off pulses set to 20% amplitude for 15 s (i.e. 15× 1 s pulses over a 30-s timespan), and the cell debris was pelleted by centrifugation at 10,000 g at 4°C for 10–20 min. The supernatant was passed through a 0.2 μm PES filter directly into a 1 mL column, with a 6 mL total capacity, containing a 0.25 mL resin bed of Streptactin sepharose (IBA, 2-1201-010) pre-washed with buffer W1. Loaded columns were washed sequentially with 0.5 mL W1 (1×) and 0.3 mL W2 (3×) (50 mM Tris-HCl, 100 mM NaCl and 5% glycerol pH 7.7) before eluting with sequential fractions of 220, 350, and 165 μL of “EB base” (25 mM Tris-HCl, 50 mM NaCl and 5% glycerol pH 7.4) supplemented with freshly added 5 mM desthiobiotin (Millipore Sigma, D1411) to form “EB.” The identification of the minor contaminant DnaK was performed via gel band excision, NH4HCO3/CH_3_CN destaining, dehydration, and subsequent MS/MS sequencing by the Penn State College of Medicine Mass Spectrometry and Proteomics Facility.

For GST-fusion proteins, cell pellets were resuspended in TBS-NoCa binding buffer (50 mM Tris-HCl, 150 mM NaCl, 1 mM MgOAc, 10 mM β-mercaptoethanol and 1 mM Imidazole, pH 8.0) and sonicated and centrifuged as above. The resultant supernatant was passed through a 0.2 μm PES filter into a 1 mL column with a 0.25 mL Pierce Glutathione Agarose (Pierce, 16100) resin bed, essentially mimicking the StrepII purification protocol. Sequential washes were performed with 2 mL (×1) and 1 mL (×2) TBS-NoCa before protein elution with sequential fractions of 220 μL (E1), 350 μL (E2), and 165 μL (E3) TBS-NoCa supplemented with 10 mM glutathione.

His-fusion proteins were purified essentially as previously described for BODIPY reactions (Maruta et al., 2019). Briefly, our purification protocol mimicked the StrepII protocol, with the following modifications: lysis/binding buffer was replaced with 15 mL of 50 mM Tris-HCl pH 8.0, 100 mM NaCl, 2 mM MgCl_2_, 0.2% C_12_E_10_, supplemented with 5 µL β-mercaptoethanol post-sonication, cell debris was pelleted by centrifugation at 30,000 g for 15 min, a 125 μL Talon (Takara, 635501) resin bed was used, the resin bed was washed with 1 mL of 50 mM Tris-HCl pH 8.0, 500 mM NaCl, 2 mM MgCl_2_, 5 mM β-mercaptoethanol, 0.2% C_12_E_10_ and 10 mM imidazole, and elution was performed with 20 mM Tris-HCl (pH 8.0), 250 mM NaCl, 5 mM β-mercaptoethanol, 10% glycerol and 250 mM imidazole.

Peak elution fractions (second eluate fraction; E2) of GST and His tagged proteins were subjected to buffer exchange using Amicon Ultra 0.5 mL 10 kDa cutoff columns (Millipore Sigma, UFC501024) with five sequential rounds of concentration performed by centrifugation at 14,000 g and 4°C for approximately 10 min and dilution with “EB base” (5×, 5×, 5×, 5×, 2×) for a total dilution of 1250×.

Protein quality and quantity were evaluated immediately after elution by SDS-PAGE of 10–20 μL fractions with a 3–4 lane mass ladder of Fraction V bovine serum albumin (BSA) (e.g., 0.5, 1.0, 1.5, 2.0 μg/lane) followed by Gel-Code Blue (Thermo Fisher Scientific, 24592) staining. Gels were imaged in a ChemiDoc MP Imaging System (Bio-Rad) and band intensity calculated using ImageLab software (Bio-Rad). Biochemical assays were initiated on the fraction displaying peak yield (almost always E2) immediately after PAGE analysis, generally 2–3 h post-elution, during which time proteins had been stored on ice in a 4°C refrigerator. We note that, under routine conditions and if pre-quantification of exact yield is not critical, the StrepII-tag E2 purity and concentration is consistent enough to allow for immediate biochemical analysis, within minutes of elution. Typically, individual StrepII-GPA1 purifications yielded enough protein for 40–50 wells in BODIPY assays when conducted in a 96-well plate format.

### 2.4 BODIPY assays

**FIGURE 1:**
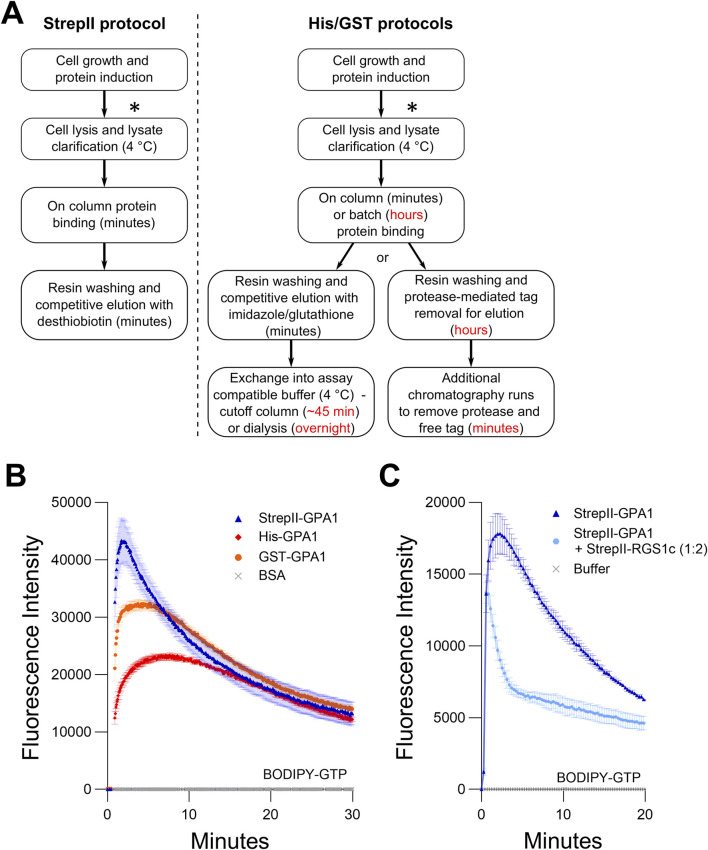
StrepII-tagged protein displays higher enzymatic activity than GPA1 proteins purified using other methods. **(A)** Comparison of the purification schemes of StrepII-GPA1 vs His-/GST-GPA1. Red text indicates points at which timing is extended in traditional protocols. The asterisks (*) indicate a potential stopping point at which cell pellets can be frozen and stockpiled for future purifications. **(B)** Comparison of BODIPY-GTP binding and hydrolysis of GPA1 isolated by StrepII-, His- and GST-tag purification procedures. The comparison between StrepII-GPA1 and His-GPA1 was performed five times, while GST-GPA1 was included twice, as the low yield of GST-GPA1, shown in Supplementary Table S3, precluded extensive characterization. **(C)** BODIPY-GTP binding and hydrolysis data for StrepII-GPA1 ± StrepII-RGS1 (cytosolic domain - StrepII-RGS1c). In **(B)** 100 nM protein was used, with bovine serum albumin (BSA) at equimolar concentration as a negative control. In **(C)** 90 nM StrepII-GPA1 was used ± 180 nM StrepII-RGS1c. The assay presented in **(C)** was performed six times with similar results; each instance utilized independently purified proteins. To generate the depicted fluorescence intensity values, the raw data values of two **(C)** or three **(B)** technical replicates for each protein were baseline-corrected using the mean replicate values for the BSA negative control **(B)** and averaged, therefore the BSA trace appears near the *x*-axis. **(C)** was treated similarly using a buffer control as the negative control. The data are graphically presented as the mean ± SEM and values below zero are not plotted.

BODIPY-GTP (BODIPY FL GTP, #G12411) and BODIPY-GTPγS (BODIPY™ FL GTP-γ-S, #G22183) stocks were purchased from Thermo Fisher Scientific and diluted to 100 nM in Tris-HCl pH 7.4 immediately prior to use. BSA or buffer alone was used as a negative control as indicated in each assay. Proteins and MgCl_2_ were diluted to twice the final assay concentration, generally 200 nM (GPA1, GNAO1 or BSA) or 400 nM (GNAI1 or BSA) supplemented with 10 mM MgCl_2_ in “EB base” on ice, normally in a master mix sufficient to perform reactions in triplicate. 100 μL of each diluted protein was aliquoted to wells of a Costar 96 well plate (Corning, 3631 - black with clear flat bottom non-treated plate) and loaded into a Synergy Neo2 multimode reader (Biotek), or in Figure 1C, an Flx800 plate reader (Biotek), set at 25°C. Pre-injection background readings were taken with monochromators set to 486/18 nm excitation and 525/20 nm emission with a gain setting within the range 90–100 (Synergy Neo2), or 485/20 nm excitation and 528/20 nm emission filters with the sensitivity set to 90 (Flx800). Reactions were initiated utilizing plate reader injectors to dispense 100 μL of BODIPY-GTP or BODIPY-GTPγS to each well (at a rate of 250 μL/s), yielding a final assay concentration of 50 nM BODIPY-GTP/-GTPγS, 100 or 200 nM protein and 5 mM Mg^2+^ cofactor. Kinetics were normally monitored in “plate mode” for 30 min with a kinetic interval of 3–6 s (Synergy Neo2) or 25–30 s (Flx800). For rapid monitoring of initial BODIPY-GTPγS binding rates, samples were monitored in “well mode” in which each well was measured with an 80 msec kinetic interval for 30 s before sequentially moving to the next well (Synergy Neo2).

### 2.5 SYPRO orange assays

We adapted the protein unfolding assay of Biggar et al. (2012) to assess protein stability over time at 25°C. Protein was diluted to 400 nM or 600 nM in “EB Base” supplemented with 5 mM MgCl_2_ and nucleotides as indicated with 5× SYPRO Orange dye (Thermo Fisher Scientific #S6650-5000× stock). Forty μl per reaction was aliquoted into wells of a FLUOTRAC 200 96 well half area plate (Greiner Bio-One, 675076), which was loaded into a Synergy Neo2 multimode reader (Biotek). Fluorescence was monitored for the indicated length of time with monochromators set to 470/20 nm excitation and 570/20 nm emission with a gain setting of 100 and a kinetic interval of 5–10 s. For assays that included a refolding component, Synergy Neo2 injectors were used for the addition of 10 µM GDP (Millipore Sigma, G7127) or 10 µM GTPγS (Millipore Sigma, 10220647001) at the indicated time points.

### 2.6 Data analysis

**FIGURE 2:**
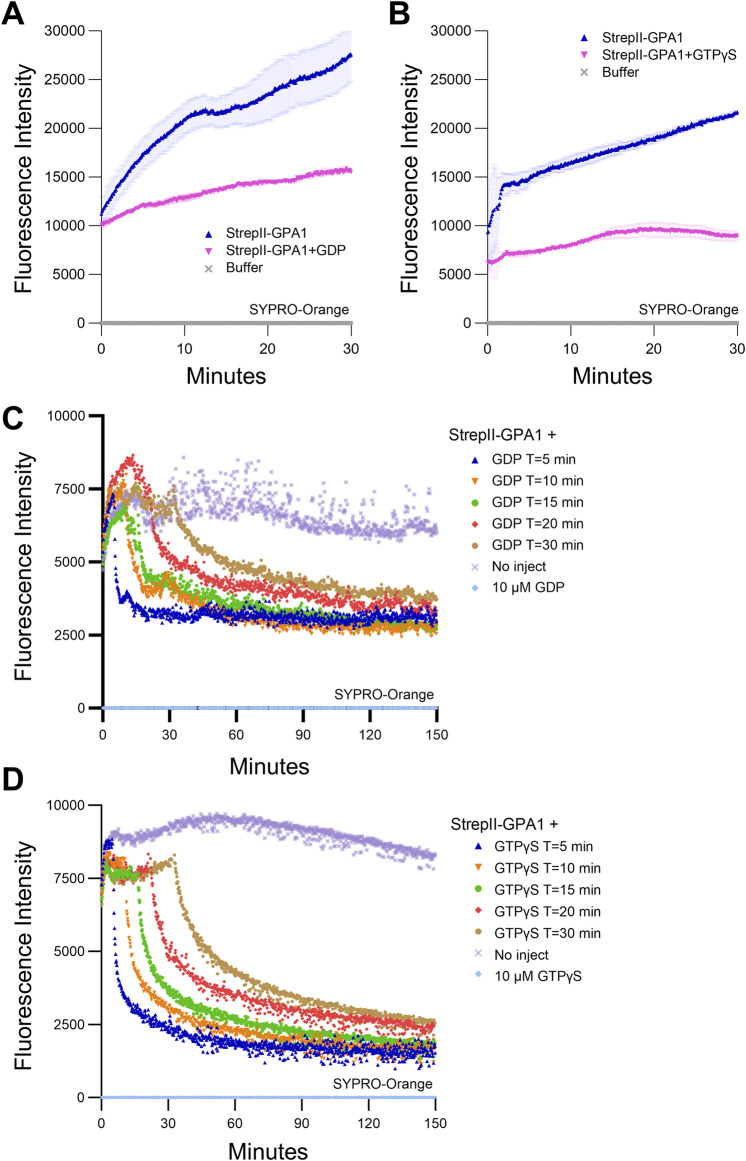
GPA1 displays temperature- and time-dependent loss of activity. **(A, B)** SYPRO Orange protein unfolding assays on GPA1 conducted at 25°C in the absence of additional nucleotides, compared to GPA1 supplemented with **(A)** 125 μM GDP or **(B)** 125 μM GTPγS. **(C, D)** SYPRO Orange protein unfolding/refolding assays on GPA1 conducted at 25°C with **(C)** 10 μM GDP or **(D)** 10 μM GTPγS injected at T = 5, 10, 15, 20 or 30 min, compared to no injection controls. 600 nM protein was used in SYPRO Orange assays. The assay presented in **(A)** was performed three times with similar results, the assay in **(B)** seven times with similar results and the assays in **(C, D)** six and five times, respectively, with similar results; each instance utilized independently purified proteins. To generate the depicted fluorescence intensity values, the raw replicate data analyzed were baseline-corrected using the mean replicate values for buffer **(A, B)**, or 10 µM GDP **(C)**, or 10 µM GTPγS **(D)** controls, which therefore appear near the *x*-axis. The data are graphically presented as the mean ± SEM in panels **A and B**, only the means shown in **(C, D)** for clarity, and values below zero are not plotted. All traces in **(A, B)** represent the mean of two technical replicates. All traces in **(C, D)** represent the mean of three technical replicates, with the exception of the **(C)** T = 10 trace**,** for which one of the three replicates was excluded due to an abrupt artifactual mid-course rise in signal.

All samples depicted in an individual figure panel were assayed in parallel within a single plate reader run to limit handling variations. BODIPY assay results represent the average of 3 technical replicates except GNAO1^GPA1hel^ in Figure 6B, which was assayed in duplicate due to yield constraints, and the samples in Supplementary Figure S1C, which were assayed in duplicate due to the time constraints of assaying an unstable protein in well-mode. BODIPY assays were repeated 2–8 independent times (independent biological replicates). SYPRO Orange assays represent the average of 2–3 technical replicates and were repeated 2–6 independent times. Instrument-collected raw data were imported into GraphPad Prism (v10.3) for analysis, baseline corrected as indicated in each figure legend, and graphically presented as the mean ± SEM for all time points, except for those in Figures 2C,D, where error bars would obscure some trends. Biochemical reaction rates were estimated by hand-adjusting parameters for one-phase association [Y = Y0 + (Plateau-Y0) × (1−e−^*k* ×*x*^)], one-phase decay [Y = (Y0-Plateau) × e^−k × *x*^ + Plateau), and plateau with one-phase decay [Y = IF(*x* < X0, Y0, Y0 + (Plateau-Y0) × (1−e^(−k × (x−X0))^)] models and cross-checking for convergence where appropriate (Prism v10.3). First-order kinetics models were employed following the precedent of Jones et al. (2011a), Johnston et al. (2008), McEwen et al. (2001), Eberth et al. (2005), Lin et al. (2014) and Hewitt et al. (2023). The estimated biochemical rate data and goodness of fit *R* ^2^ values are available in Supplementary Table S2. Data for Figures 2C,D within a span of 5 s before and one second after nucleotide injection were subjected to outlier identification using the robust nonlinear outlier identification module provided in the Prism software, with a 1% false discovery rate. After this analysis, two time points each were removed from Figure 2C T = 20 and T = 30 assays, and 2–5 time points each were removed from the T = 15, 20, and 30 assays in Figure 2D.

## 3 Results

### 3.1 Rapid purification of recombinant GPA1 via tandem StrepII tags yields higher activity *in vitro*

The dual StrepII tag consists of tandem StrepII tags separated by a flexible linker. The original Strep tag was identified as a streptavidin binding tag that could be used to isolate recombinantly expressed antibodies (Schmidt and Skerra, 1993). Both the Strep tag as well as streptavidin were further engineered to form a StrepII-Streptactin system with increased affinity, and the option of either N-terminal or C-terminal tagging (Schmidt et al., 1996; Schmidt et al., 2013). As the resin-conjugated Streptactin used to purify StrepII-tagged proteins exists in a tetrameric state, the use of two tandem StrepII tags separated by a linker was subsequently employed to further increase binding affinity via the avidity effect while still allowing efficient competitive elution (Schmidt et al., 2013) in a buffer compatible with many downstream *in vitro* assays. These characteristics offer the advantages of rapid purification of highly pure protein without the need for post-elution processing (Figure 1A). We therefore utilized dual N-terminal StrepII tags separated by a linker sequence (SGGSGTSGGSA), similar to the linker used in the Twin-Strep-tag (GGGSGGGSGGSA) (Schmidt et al., 2013), to purify GPA1, the sole canonical Gα subunit from Arabidopsis. We adapted the base pGEX vector backbone to include N-terminal dual-StrepII tags, thrombin and TEV protease sites, a multiple cloning site, and the option of a C-terminal FLAG tag in a new vector we named pSTTa. GPA1 expressed from pSTTa was purified and observed to be highly pure (Supplementary Figure S1A). After numerous trials of multiple growth and induction protocols, we found that use of BL21 (DE3) cells cultured in high salt LB (HSLB) media resulted in the highest and most consistent yields of StrepII-GPA1, so we utilized HSLB for growth and induction in all subsequent experiments.

We first compared StrepII-GPA1 purification to existing His-GPA1 and GST-GPA1 methods (Figure 1A). To circumvent detrimental overnight dialysis steps that risk protein aggregation (Zelent et al., 2001), we prepared His-GPA1 and GST-GPA1 fusion proteins fresh and as rapidly as possible, utilizing 10 kDa molecular weight cut-off centrifugal filter units for post-elution buffer exchange into “EB Base,” the buffer used for StrepII-GPA1 elution but lacking desthiobiotin. When purified side-by-side with StrepII-GPA1, the buffer exchange steps applied to the His-GPA1 and GST-GPA1 proteins added approximately 45 min of additional handling (Figure 1A), which was performed on ice and in a 4°C refrigerated centrifuge, while the StrepII-GPA1 sample was kept on ice. When separated by SDS-PAGE, the StrepII-GPA1 protein was obviously more pure than the His-GPA1 or GST-GPA1 preparations under these rapid purification conditions (Supplementary Figure S1B). After multiple rounds of side-by-side purification it was notable that the yield of the StrepII-GPA1 protein was consistently ∼2-fold and ∼8-fold higher than that of His-GPA1 or GST-GPA1, respectively (Supplementary Table S3).

In a classic *in vitro* assay, fluorescent signal increases as Gα proteins bind BODIPY-conjugated GTP and decreases when GTP hydrolysis outpaces GTP binding, due to resultant partial fluorophore re-quenching (McEwen et al., 2001; Jameson et al., 2005; Willard et al., 2005; Maruta et al., 2019; Solis et al., 2021; Jeong and Chung, 2023). We found that freshly purified StrepII-GPA1 exhibits a characteristic BODIPY-GTP kinetic curve indicative of rapid GTP binding and rapid GTP hydrolysis (Figure 1B). Despite comparable affinity purification protocols and rapid handling at cold temperatures for the post-purification buffer exchange steps, the His-GPA1 and GST-GPA1 proteins displayed lower apparent binding (39% and 77% of the StrepII-GPA1 rate of 3.139 min^−1^) and hydrolysis (15% and 61% of the StrepII-GPA1 rate of 0.094 min^−1^) activities than the StrepII-GPA1 protein (Figure 1B; Supplementary Table S2). As peak fluorescence of BODIPY-GTP is a function of net binding and hydrolysis, another interpretation for the lower activity observed for His- and GST-fusions in Figure 1B is that GTP binding rate is unaltered between the proteins, but GTP hydrolysis is faster in the His- and GST-fusions. We therefore assayed binding to the non-hydrolyzable BODIPY-GTPγS using rapid sampling in well mode for 30 s to assess the initial relative GTPγS-binding rates. Indeed the StrepII-GPA1 protein displayed appreciably faster BODIPY-GTPγS binding compared to the His-GPA and GST-GPA1 preparations (Supplementary Figure S1C), consistent with the interpretation that the StrepII-GPA1 preparation displays higher activity.

An alternative explanation for the increased apparent activity of the StrepII-GPA1 protein compared to His-GPA1 and GST-GPA1 (Figure 1B) is that a co-purifying contaminant in the StrepII-GPA1 preparations displays GTP-binding and hydrolysis activities. Though the StrepII purifications commonly yield highly pure protein, we almost always observed a minor contaminating band slightly larger than 70 kDa in our GPA1 elutions (Supplementary Figure S1A). To verify the lack of GTP binding by any co-purifying contaminant, we expressed and purified a GPA1^S52N^ mutant that, as observed previously for a GPA1^S52C^ mutant (Maruta et al., 2019), does not bind GTP. StrepII-GPA1^S52N^ displayed no BODIPY-GTP or BODIPY-GTPγS-binding activity (Supplementary Figures S1D,E), confirming that the binding and hydrolysis observed for StrepII-GPA1 was solely a result of GPA1 activity. Mass spectrometric identification was performed on the ∼70 kDa protein and it was found to correspond to *E. coli* DnaK, which is not expected to display GTP binding activity (Barthel and Walker, 1999).

We further functionally validated our StrepII-GPA1 protein using RGS1, which is a key biochemical regulator of GPA1. Arabidopsis RGS1 encodes a protein with seven transmembrane spanning domains at the N-terminus and a cytosolic RGS domain at the C-terminus that stimulates the GTPase activity of GPA1. Previous studies of RGS1 activity have therefore utilized only the cytosolic RGS domain to study its effect on GPA1 *in vitro* (Willard and Siderovski, 2004; Jones et al., 2011b). We recombinantly produced the RGS1 cytosolic domain utilizing the pSTTa vector and tested its effect on StrepII-GPA1 to confirm: 1) that RGS1 is also amenable to rapid purification via dual StrepII-tags, and more importantly; 2) that the StrepII-tagging approach for GPA1 did not disrupt GPA1 interaction with the primary regulator, RGS1. The addition of StrepII-RGS1 to StrepII-GPA1 in a BODIPY-GTP assay strongly promoted the hydrolysis of GTP by GPA1 (Figure 1C), indicating both 1 and 2 are true.

We note that when our assays were run in well mode, the variation between technical replicates of the StrepII-GPA1 samples was high with a drop in signal observed from wells measured later within the same run (Supplementary Figure S1F), as reflected by the large error bars in Supplementary Figures S1C,E. To establish if this variability was intrinsic to StrepII-GPA1, or an artifact of the delay between measurement of samples in a well mode assay, we performed a well mode assay in which three StrepII-GPA1 technical replicates were measured sequentially, prior to the negative control reactions (Supplementary Figure S1G). This rapid sampling resulted in greatly reduced replicate-to-replicate variability as displayed by the smaller error bars in Supplementary Figure S1G compared to Supplementary Figure S1C. These results also were consistent with a time-dependent loss of activity for GPA1, a phenomenon we investigated further through assessment of GPA1 stability.

### 3.2 GPA1 stability

To investigate the underlying cause of *in vitro* GPA1 activity loss observed during a sampling time course (Supplementary Figure S1F), we assessed StrepII-GPA1 conformational stability utilizing SYPRO Orange fluorescence. SYPRO Orange fluorescence increases upon interaction with hydrophobic regions of a protein, which will have greater accessibility upon protein unfolding. We observed that at our standard BODIPY-GTP binding assay temperature of 25°C, GPA1 protein displayed a steady increase in SYPRO Orange fluorescence over the course of 30 min, indicative of protein unfolding (Figures 2A,B) as the cause of the activity loss. This increase in protein instability was largely counteracted by incubating GPA1 with excess unlabeled GDP (Figure 2A) or GTPγS (Figure 2B). Indeed, many Gα purification protocols include excess concentrations of GDP in buffers to stabilize the protein and prevent aggregation (Zelent et al., 2001) during protracted purification protocols such as those that utilize proteolytic cleavage or buffer exchange by techniques including overnight dialysis [(Lee et al., 1994; Wall et al., 1995; Thomas et al., 2008; Jones et al., 2011a; Urano et al., 2012; Kataria et al., 2013; Lyon et al., 2013; Dror et al., 2015; Kant et al., 2016; Maruta et al., 2019; Knight et al., 2021; Hewitt et al., 2023) and Figure 1A]. We predicted that, given the greatly reduced processing time, our streamlined StrepII purification protocol would be suitable for purification of active Gα proteins without the need for supplementation with excess GDP. Gα proteins purified from cell lysates are expected to remain at least partially GDP loaded in the short term from production in *E. coli*, however, over time if not provided with excess nucleotide, GDP dissociation will result in protein destabilization. Indeed, when assayed fresh our StrepII-GPA1 preparations displayed higher peak signals and increased BODIPY-GTPγS (Supplementary Figure S2A; Supplementary Tables S2, S4) and BODIPY-GTP (Supplementary Figure S2B; Supplementary Tables S2, S4) binding activities compared to preparations that had been freshly eluted in the presence of 10 µM GDP. These data confirmed that when prepared with our rapid purification protocol and assayed immediately post-quantification, StrepII-GPA1 could be assayed without GDP supplementation.

To correlate GPA1 unfolding with loss of activity over time in the absence of excess nucleotides, we assayed BODIPY-GTP binding with or without a 30-min 25°C preincubation, analogous to the SYPRO-Orange assay timeline in Figure 2A. After the 30-min 25°C preincubation GPA1 displayed clearly reduced BODIPY-GTP binding activity (Supplementary Figure S2C), with an association rate (2.123 min^−1^) only 57% that of freshly eluted StrepII-GPA1 (3.706 min^−1^) (Supplementary Table S2). The inclusion of excess (10 µM) GDP, with or without 30-min preincubation at 25°C, reduced maximum net binding signals by ∼75% (Supplementary Figure S2C; Supplementary Table S4). Therefore, the presence of excess (10 μM) GDP prevented temperature-dependent loss of activity (Figures 2A,B); however, competition assays revealed that GDP also suppressed BODIPY-GTP binding (Supplementary Figures S2A–C), which could lead to underestimates of binding and hydrolysis rates.

We then investigated the extent to which nucleotides could stimulate GPA1 refolding. SYPRO Orange assays at 25°C were performed as before, except GDP or GTPγS were injected at different time points during the first 30 min. Notably, with multiple samples assayed in triplicate the setup time of the assay took several minutes, and by the time measurements were initiated it is presumed some portion of the early unfolding phase displayed in Figures 2A,B had occurred. Addition of GDP (Figure 2C) or GTPγS (Figure 2D) during the course of the assay stimulated a partial reduction in SYPRO Orange signal, indicative of protein refolding induced by the nucleotide. The reduction in SYPRO Orange signal was generally more rapid and occurred at a greater magnitude when the nucleotides were injected at earlier time points, e.g., T = 5 min vs. T = 30 min (Supplementary Table S2). These results indicate that GPA1 unfolding is partially reversible by nucleotide addition, but the extent and speed to which GPA1 can refold is time-dependent.

We next examined if StrepII-GPA1 was amenable to storage overnight at 4°C. Fresh StrepII-GPA1 (Supplementary Figure S2D) displayed 19% greater maximal GTP binding, 46% faster binding kinetics, and a 66% faster hydrolysis rate compared to StrepII-GPA1 after overnight storage at 4°C (Supplementary Tables S2, S4), indicating that overnight storage causes an overall loss of activity. Given the stability issues of GPA1 outlined above, we hypothesized that storage at −80°C and freeze-thawing also has an impact on activity of GPA1 in the absence of GDP supplementation. Therefore, we purified StrepII-GPA1, aliquoted the preparation into various concentrations of either glycerol or sucrose as a cryoprotectant (Hauptmann et al., 2018), snap froze these fractions with liquid N_2_ and stored the proteins for 3 weeks at −80°C. Upon thawing and assaying the protein, we observed that GPA1 frozen with sucrose as a cryoprotectant displayed higher peak BODIPY-GTP binding activity than GPA1 frozen with glycerol (Supplementary Figure S3A). We then repeated the assay to compare the binding and hydrolysis curves of StrepII-GPA1 protein stored with the optimal sucrose concentration, 8.3%, vs. the optimal glycerol concentration, 10%. Supplementary Figure S3B shows StrepII-GPA1 frozen with sucrose exhibited 30% more rapid BODIPY-GTP binding and 123% faster hydrolysis compared to StrepII-GPA1 stored with glycerol (Supplementary Table S2). StrepII-GPA1 prepared either fresh or frozen with 8.3% sucrose displayed similar BODIPY-GTP binding and hydrolysis curve shapes (Figure 1B vs. Supplementary Figure S3B). This difference suggests the use of glycerol as a cryoprotectant could result in an underestimate of the peak net hydrolysis rate of GPA1 preparations.

With the exception of the frozen storage experiments reported above, all of the data presented herein are results from freshly isolated protein. In the following comparative experiments, freshly isolated GPA1, GNAI1, and GNAO1 proteins were stored on ice in a 4°C refrigerator during post-elution quantification steps, the reaction mixes were prepared on ice, and once aliquoted to plates were subjected to no more than 2 min of temperature equilibration immediately prior to initiating assays, to mitigate loss of activity.

### 3.3 Comparison of GPA1 to human GNAI1/GNAO1

Arabidopsis GPA1 bears high structural similarity to human GNAI1, which has provided a rationale for previous biochemical comparisons of GPA1 with GNAI1 such as those conducted by Jones et al. (2011a). We therefore sought to extend our newly optimized recombinant Gα purification protocol to GNAI1. We cloned GNAI1 into pSTTa using both codon harmonized (GNAI1ch) and wild-type (GNAI1wt) sequence (Supplementary Figures S4A,B). We found that proteins derived from the two constructs were essentially interchangeable as side-by-side comparisons showed the GNAI1wt and GNAI1ch proteins did not differ in yield or purity (Supplementary Figure S4C), in BODIPY-GTP binding and hydrolysis (Supplementary Figure S4C), or in BODIPY-GTPγS binding (Supplementary Figure S4C). To expand upon the previous in-depth comparison of GPA1 with GNAI1 (Jones et al., 2011a), we prepared a construct for another human Gα_i_ subfamily member, GNAO1. GNAO1 has been shown to display considerably faster kinetics than GNAI1 (Solis et al., 2021) and in a brief comparison to GPA1, displayed more similar kinetic properties to GPA1 (Johnston et al., 2007) than GNAI1 did. On a sequence level, the GNAI1 protein shares 38.2% identity and 56.8% similarity with GPA1, while GNAO1 displays 37.0% identity and 54.1% similarity with GPA1. Therefore, it is reasonable to compare GPA1 to both of these mammalian Gα_i_ proteins.

**FIGURE 3:**
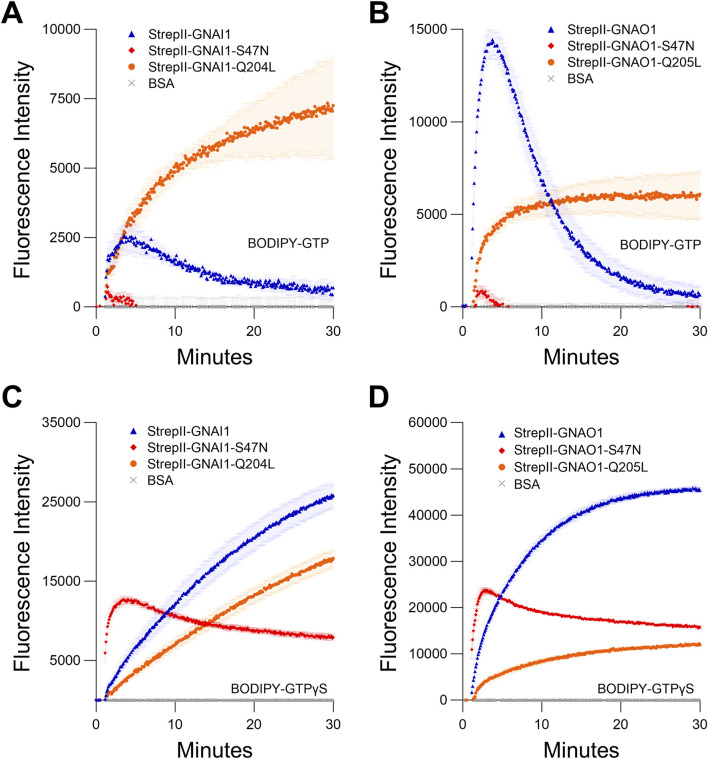
Comparison of StrepII-GNAI1 and StrepII-GNAO1 to their dominant negative (S47N) and constitutively active (Q204L/Q205L) mutants. **(A, B)** BODIPY-GTP binding and hydrolysis curves of **(A)** GNAI1 or **(B)**. GNAO1, with corresponding mutants. **(C, D)** BODIPY-GTPγS binding curves of **(C)** GNAI1 or **(D)** GNAO1, with corresponding mutants. Note: for all graphs, wild-type = blue, S47N = red, and Q204L/Q205L = orange. The assays presented in this figure were all performed twice using independently purified proteins with similar results. To generate the depicted fluorescence intensity values, the raw data values of three technical replicates for each protein were baseline-corrected using the mean replicate values for the BSA negative control and averaged, therefore the BSA traces appear near the *x*-axes. The data are graphically presented as the mean ± SEM and values below zero are not plotted.

We purified StrepII-GNAI1 and StrepII-GNAO1, and compared their wild-type activities to those of their constitutively active mutants (StrepII-GNAI1^Q204L^/GNAO1^Q205L^ (Kleuss et al., 1994)), and to activities of mutants (StrepII-GNAI1^S47N^/StrepII-GNAO1^S47N^) corresponding to the plant nucleotide-free (Supplementary Figures S1D,E) GPA1^S52N^ mutant. Both wild-type GNAI1 and GNAO1 proteins displayed GTP-binding and hydrolysis activities, as expected based on previous studies (Solis et al., 2021). The net GTP binding activity of GNAI1, reflected by the amplitude of peak fluorescence, was considerably lower (5.6-fold) than that of GNAO1 (Figures 3A,B; Supplementary Table S4). These results demonstrate the StrepII purification protocol is applicable to human Gα subunits. The constitutively active mutants (Q204L/Q205L) displayed slower binding than the wild-type proteins and no hydrolysis activity, as expected (Graziano and Gilman, 1989; Kleuss et al., 1994; Wu et al., 2018; Maruta et al., 2019), while the S47N mutants displayed no BODIPY-GTP binding activity (Figures 3A,B), in agreement with the results of Knight et al. (2023). Surprisingly, the S47N mutants both displayed BODIPY-GTPγS binding activity with initial rates that were faster than was observed for the wild-type GNAI1 and GNAO1 proteins (Figures 3C,D). The binding activity, however, showed a peak at 3–4 min, followed by a steady decline in signal. Given that the BODIPY fluorophore is covalently attached differently in BODIPY-GTP (ribose ring) and BODIPY-GTPγS (γ-phosphate), inconsistencies in binding results between the two BODIPY reagents could arise from a combination of steric differences of the binding pocket between S47N mutants and the respective locations of the BODIPY fluorophore.

The steady decline of BODIPY-GTPγS binding by the GNAI1 and GNAO1 S47N mutants is unlikely to be due to hydrolysis as GTPγS is considered non-hydrolysable, and no evidence of BODIPY-GTPγS hydrolysis was evident in any of our assays with wild-type GNAI1 or GNAO1. These results are in contrast to the analogous GPA1^S52N^ mutant, which displayed no BODIPY-GTPγS binding (Supplementary Figure S4E). The S47 residue within the G1 motif is important for Mg^2+^ cofactor coordination in Gα subunits (Wall et al., 1998) and small monomeric G proteins carrying mutations at the equivalent site have also been shown to display impaired Mg^2+^ coordination and affinity (Farnsworth and Feig, 1991; John et al., 1993). Since other metal ions are known to inhibit Gα nucleotide binding (Gao et al., 2005), we routinely utilized trace-metal-free (TMF) grade components to standardize our assays, which explicitly ruled out any effect of extraneously-present divalent ions, including Mg^2+^. Notably, we also show that our methodology can be performed using standard grade reagents (Supplementary Figure S4F). To examine if the S47N mutants do retain some residual Mg^2+^ binding, we performed BODIPY-GTPγS binding assays ± 5 mM Mg^2+^. In our ± Mg^2+^ assay, both wild-type GNAI1 and GNAO1 and S47N mutants of GNAI1 and GNAO1 displayed a clear requirement for Mg^2+^, with a very low level of BODIPY-GTPγS binding activity observed in the absence of Mg^2+^ (Supplementary Figures S4G,H; Supplementary Table S4). These data suggest GNAI1^S47N^ and GNAO1^S47N^ do retain some affinity for Mg^2+^ and a requirement for this cofactor in nucleotide coordination.

**FIGURE 4:**
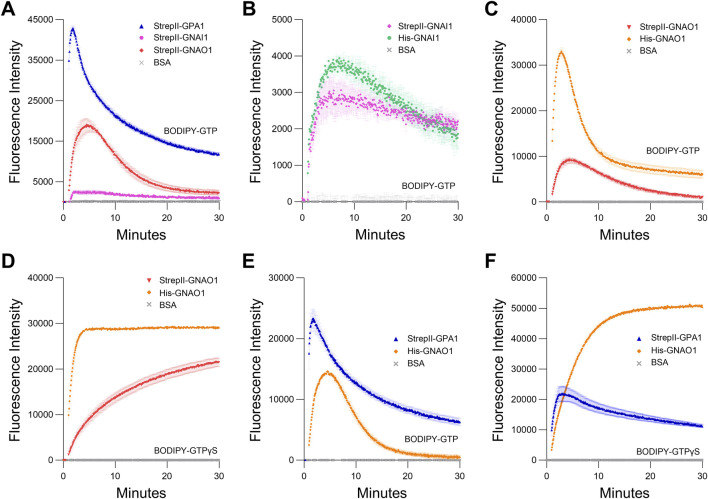
Comparison of GPA1 activity to GNAI1 and GNAO1 activity. **(A)** BODIPY-GTP binding and hydrolysis curves of StrepII-GPA1, StrepII-GNAI1 and StrepII-GNAO1. **(B, C)** BODIPY-GTP binding and hydrolysis curves of **(B)** StrepII-GNAI1 vs His-GNAI1 and **(C)** StrepII-GNAO1 vs His-GNAO1. Note that the low activity of GNAI1, as evident in **(A)**, results in a *y*-axis scale difference for **(B)** that increases the visibility of noise and minor differences in GNAI1 activity. **(D)** BODIPY-GTPγS binding curves of StrepII-GNAO1 vs His-GNAO1. **(E, F)** Comparison of enzyme kinetics of StrepII-GPA1 vs His-GNAO1. **(E)** Binding and hydrolysis of BODIPY-GTP or **(F)** binding of BODIPY-GTPγS. Gα proteins were used at 200 nM protein in **(B)** and 130 nM protein in **(C, D)**, while 100 nM protein was used in **(A, E, F)** The assays presented in this figure were performed two or three times using independently purified proteins with similar results. To generate the depicted fluorescence intensity values, the raw data values of three technical replicates for each protein were baseline-corrected using the mean replicate values for the BSA negative control and averaged; therefore, the BSA traces appear near the *x*-axes. The data are graphically presented as the mean ± SEM and values below zero are not plotted.

Next, we compared the binding activities of StrepII-GPA1 to StrepII-GNAI1 and StrepII-GNAO1. When compared to StrepII-GPA1, StrepII-GNAI1 displayed a much lower apparent BODIPY-GTP binding peak (6% of StrepII-GPA1), while StrepII-GNAO1 displayed an intermediate activity (44% of StrepII-GPA1) (Figure 4A; Supplementary Table S4). As peak fluorescence reflects the net activity of GTP binding and hydrolysis, these initial results were consistent with GPA1 displaying a faster binding and slower hydrolysis rate than GNAI1. GNAO1 displayed greater net BODIPY-GTP binding compared to GNAI1, and an activity more similar to that of GPA1. As the StrepII purification protocol was superior to His purification for GPA1, we characterized StrepII-tagged GNAI1 and GNAO1 in comparison to the commonly used His-tagged GNAI1 and His-tagged GNAO1. We observed that different Gα subunits, even within the Gαi family, display their own optimal tag and purification method. Minor differences in activity were observed between His-GNAI1 and StrepII-GNAI1 (Figure 4B), indicating that StrepII or His purification is suitable for GNAI1. By contrast, His-GNAO1 displayed higher net BODIPY-GTP binding (1.8-fold) and hydrolysis activities (2.9-fold) than StrepII-GNAO1 (Figure 4C; Supplementary Table S2), and the difference was similarly evident for binding of BODIPY-GTPγS (Figure 4D), indicating the His purification protocol is the more suitable method to assay GNAO1 activity.

**FIGURE 5:**
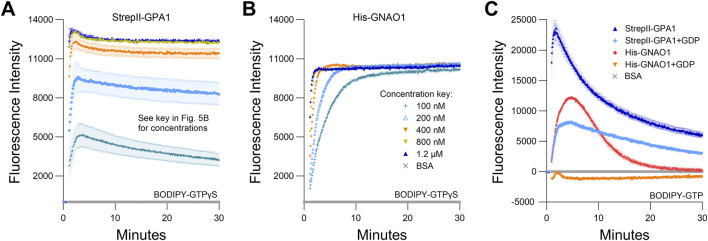
Saturation of BODIPY-GTPγS binding occurs at lower protein concentrations for GNAO1 than GPA1. **(A, B)** Concentration-dependent kinetics and maximal binding of 50 nM BODIPY-GTPγS by **(A)** StrepII-GPA1 or **(B)** His-GNAO1. **(C)** Comparison of binding and hydrolysis of BODIPY-GTP by StrepII-GPA1 vs His-GNAO1 ± 10 µM GDP. Gα proteins were used at 100 nM protein in panel **(C)**. The assays presented in this figure were performed two or three times using independently purified proteins with similar results. To generate the depicted fluorescence intensity values, the raw data values of two **(A, B)** or three **(C)** technical replicates for each protein were baseline-corrected using the mean replicate values for the BSA negative control and averaged, therefore the BSA traces appear near *y* = 0. The data are graphically presented as the mean ± SEM and values below zero are not plotted, except in **(C)** to show the presence of the His-GNAO1 + GDP samples.

Given the above results, we performed side-by-side purifications of StrepII-GPA1 and His-GNAO1, i.e., utilizing the optimal tag choice for each protein, between those compared above, to yield the most valid comparison between proteins. We demonstrate that GPA1 does indeed display a faster GTP binding rate than GNAO1 (3.25 vs. 0.80 min^−1^), but the net hydrolysis rates appear not to be as different between plant and human Gα subunits as previously thought (Johnston et al., 2007; Johnston et al., 2008; Jones et al., 2011a), with His-GNAO1 displaying just a 1.8-fold higher hydrolysis rate than StrepII-GPA1 (Figure 4E; Supplementary Table S2). To isolate the observed binding rate of the Gα proteins, we performed assays with the non-hydrolyzable GTP analog BODIPY-GTPγS. Indeed StrepII-GPA1 displayed a 7-fold higher initial rate of BODIPY-GTPγS binding than His-GNAO1 (Figure 4F; Supplementary Table S2). Contrastingly, the StrepII-GPA1 maximal BODIPY-GTPγS binding signal was 2.3-fold lower than that of His-GNAO1, and rather than plateauing as with GNAO1, the GPA1 BODIPY-GTPγS signal peaked followed by a decrease over time (Figure 4F; Supplementary Table S4). Possible reasons for the difference in signal maxima include: 1) steric differences of the Gα binding pockets resulting in differential levels of BODIPY fluorophore unquenching upon protein binding, and; 2) inherent instability of GPA1 resulting in a lower apparent binding activity *in vitro*. We believe hypothesis 1) is unlikely as the empirically derived crystal structures of GPA1 and GNAO1 are highly similar (Supplementary Figure S5A), just as are the structures of GPA1 and GNAI1 (Supplementary Figure S5B). We therefore sought to assess the amount of each enzyme necessary to observe saturated and stable binding of 50 nM BODIPY-GTPγS, as a reflection of Gα activity retained *in vitro*. Despite being in excess, 100 nM, 200 nM and 400 nM concentrations of StrepII-GPA1 were unable to attain a maximal binding signal with 50 nM BODIPY-GTPγS. Only 800 nM or 1.2 µM GPA1 displayed a stable binding plateau at the maximal level, with association binding rates of 3.364 and 3.531 min^−1^, respectively (Figure 5A; Supplementary Table S2). In comparison, all concentrations of GNAO1 either attained a maximal plateau, or neared maximal fluorescence in the case of 100 nM GNAO1, within the course of our assay (Figure 5B; Supplementary Table S4). The necessity for higher GPA1 concentrations in reaching binding saturation reflects the lower stability of GPA1 *in vitro* (Figures 2A,B) compared to GNAO1 and GNAI1 (Supplementary Figure S5C), but also provides insight regarding the GNAO1>GPA1 signal maxima in Figure 4F. Notably, the maximal levels of BODIPY-GTPγS fluorescence were similar between high concentrations of GPA1 and GNAO1 when assayed side-by-side (Figures 5A,B), thereby indicating that steric differences in the binding pockets do not result in markedly different levels of BODIPY fluorophore unquenching and thus refuting hypothesis 1) above. Next we compared the ability of excess (10 µM) GDP to suppress binding of 50 nM BODIPY-GTP to 100 nM Gα protein. Peak BODIPY-GTP binding was suppressed 2.9-fold by 10 µM GDP for GPA1; by contrast, BODIPY-GTP binding was almost completely abolished for GNAO1 by 10 µM GDP (Figure 5C; Supplementary Table S4). The striking difference in GDP suppression of GTP binding likely reflects a higher relative affinity for GTP than GDP of GPA1, as previously reported (Johnston et al., 2007), and a significantly faster nucleotide exchange rate of GPA1 than GNAO1.

### 3.4 GPA1-GNAO1 helical domain swaps

**FIGURE 6:**
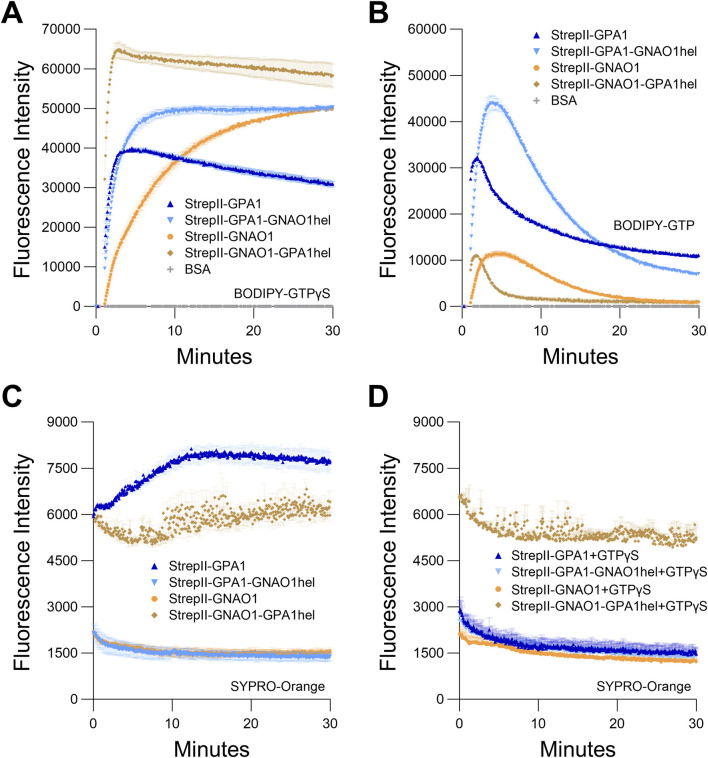
Helical domain swap between GPA1 and GNAO1. Regions encoding the helical domains of GPA1 (residues 68–188) and GNAO1 (residues 63–177) were reciprocally swapped by overlap-extension PCR and the resultant constructs were all expressed with dual StrepII-tags to eliminate the tag as a variable. **(A, B)** Curves of GPA1, GNAO1 and helical domain swaps for **(A)** BODIPY-GTPγS binding and **(B)**. BODIPY-GTP binding and hydrolysis. **(C, D)** SYPRO Orange protein unfolding assays conducted at 25°C with GPA1, GNAO1, GPA1^GNAO1hel^ or GNAO1^GPA1hel^ **(C)** in the absence of supplementation with additional nucleotides, or **(D)** in the presence of 10 µM GTPγS. 100 nM protein was used in BODIPY assays and 400 nM protein in SYPRO Orange assays. The assays presented in this figure were performed two or three times using independently purified proteins with similar results. To generate the depicted fluorescence intensity values, the raw data values of two or three technical replicates for each protein were baseline-corrected using the mean replicate values for the BSA negative control and averaged, therefore the BSA traces appear near the *x*-axes in panels **(A, B)**, and values below zero are not plotted.

It has been previously demonstrated that the helical domain of GPA1 displays a marked level of intrinsic disorder and increased dynamic motion compared to that of GNAI1 (Jones et al., 2011a). Jones et al. (2011a) confirmed that a helical domain swap between GPA1 and GNAI1 largely swapped the relative kinetics between the 2 Gα proteins. In those studies, the GPA1 helical domain conferred rapid spontaneous activation to GNAI1 while the GNAI1 helical domain conferred slower activation to GPA1. Given the more similar activities of GPA1 and GNAO1 (*cf.* Figure 4E), we sought to expand upon these analyses to assess if a helical domain swap between GPA1 and GNAO1 would: 1) display as strong a difference as the GPA1-GNAI1 domain swap, and 2) confirm that the helical domain of GPA1 is responsible for the poor stability of GPA1. Therefore, we created reciprocal domain swap constructs GPA1^GNAO1hel^ (GPA1 Ras domain fused to the GNAO1 helical domain) and GNAO1^GPA1hel^ (GNAO1 Ras domain fused to the GPA1 helical domain). To not confound any tag/purification effects with the domain swap effects, we utilized our StrepII tagging and purification methods for all proteins, and similar to our wild-type and point mutant proteins, all domain-swapped protein purifications displayed excellent post-purification integrity, though the yield of the GNAO1^GPA1hel^ protein was notably lower than that of other proteins (Supplementary Figure S6). A comparison of BODIPY-GTPγS binding demonstrated that binding rates increased in the following order: GNAO1 < GPA1^GNAO1hel^ < GPA1 < GNAO1^GPA1hel^ (Figure 6A; Supplementary Table S2). Beyond this initial binding rate, GPA1 displayed the lowest signal amplitude corresponding to peak binding, while GNAO1^GPA1hel^ displayed the highest signal plateau (Figure 6A; Supplementary Table S4). In BODIPY-GTP assays, which integrate GTP binding and hydrolysis, a similar initial trend was largely displayed during the binding phase: GNAO1 < GPA1^GNAO1hel^ < GNAO1^GPA1hel^ < GPA1 (Figure 6B; Supplementary Table S2). Once BODIPY-GTP hydrolysis exceeded the binding rate, we observed the following order of maximal net hydrolysis rates: GNAO1 = GPA1^GNAO1hel^ < GPA1 < GNAO1^GPA1hel^ (Figure 6B; Supplementary Table S2). Therefore, although the rate of BODIPY-GTPγS binding by GNAO1^GPA1hel^ was the fastest of the four proteins assayed, and rapid binding of BODIPY-GTP by GNAO1^GPA1hel^ was also evident in the initial seconds of the assay, its peak BODIPY-GTP fluorescent signal was dampened by a rapid switch to net hydrolysis. We then assessed the relative conformational stability of the GPA1, GNAO1, GPA1^GNAO1hel^ and the GNAO1^GPA1hel^ proteins in a SYPRO Orange assay ± 10 µM GTPγS (no BODIPY label). As suspected, the GNAO1 helical domain conferred a similar stability to GPA1 as did excess GTPγS (Figures 6C,D). As before, GPA1 samples without nucleotide supplementation displayed increased SYPRO Orange signal indicative of protein unfolding, and GPA1 was the only protein in the domain swap assays to display considerable divergence between the ± GTPγS samples (Figures 6C,D). Interestingly, at “T = 0” of the SYPRO Orange assay, the fluorescence in the absence of nucleotide supplementation was already much higher for GPA1 than for GNAO1 or GPA1 supplemented with GTPγS (Figures 6C,D). We note that all of these samples were prepared on ice in duplicate, pipetted into the assay plate and loaded into the plate reader; a process that took ∼4 min for the number of samples in Figures 6C,D. To investigate the difference at “T = 0” of the assays comparing multiple samples, we performed a 1 vs. 1 assay comparing single wells of GPA1 vs GPA1 + 10 µM GDP. Assays of such a small number of samples can be initiated in seconds and this allowed us to monitor SYPRO Orange fluorescence almost immediately after removal from ice. Indeed, in this rapid assay, the initial fluorescence levels were similar between the samples before a steady rise in fluorescence signal was observed in the GPA1 alone reaction (Supplementary Figure S5D). The initial similarity of fluorescence between ± nucleotide samples was quite similar to the results shown in Figures 2A,B, which were assays run on an intermediate scale compared to the large assays in Figures 2C,D, 6C,D, and the small scale assay in Supplementary Figure S5D. GNAO1^GPA1hel^ in the large scale assay also displayed a higher initial value of SYPRO Orange fluorescence, and noticeably more signal variation between time points, though it should be noted that this noise-like variation was not always observed for GNAO1^GPA1hel^ (Supplementary Figure S5E). Unlike GPA1, the addition of GTPγS did not repress the T = 0 high fluorescence values for GNAO1^GPA1hel^, yet the fluorescence signals for GNAO1^GPA1hel^ did not rise as rapidly through the assay as they did for GPA1 in the absence of GTPγS (Figures 6C,D). These traits appear consistent with GNAO1^GPA1hel^ achieving a stable but different conformation than the other Gα proteins assayed in Figure 6; a conformation that is seemingly characterized by increased surface accessibility of hydrophobic residues for SYPRO Orange binding, but with strong BODIPY-GTPγS binding as evident in Figure 6A, Supplementary Tables S2, S4, indicative of a highly active protein. A decrease in BODIPY-GTPγS fluorescence for GNAO1^GPA1hel^ was observed after peak signal had been reached (Figure 6A), indicating a loss of activity over the course of the assay, similar to the loss observed for GPA1. However, wild-type GPA1 displayed a lower peak of BODIPY-GTPγS fluorescence, indicating a more rapid loss of activity for GPA1 than GNAO1^GPA1hel^, consistent with the more immediate unfolding observed for GPA1 in Figure 6C. In summary, for GPA1 *in vitro*, unfolding at room temperature during plate setup and then at 25°C upon assay initiation in the plate reader began almost immediately and was evident on a scale of seconds to minutes (Supplementary Figure S5D), further underscoring the need to use a rapid purification protocol. Interestingly, our domain swap assays indicate that neither the Ras nor the helical domain alone accounts for the full lack of GPA1 stability, as the magnitude of BODIPY-GTPγS binding indicates the GPA1 helical domain does not destabilize GNAO1 as rapidly as it does GPA1.

## 4 Discussion

Purification of functional recombinant heterotrimeric G protein Gα subunits is integral to understanding their roles in both animals and plants. The former is of importance due to their well-described functions in human health (Weinstein and Shenker, 1993), and the latter is important due to G protein involvement in controlling agriculturally important traits (Botella, 2012; Ferrero-Serrano et al., 2024). We demonstrate for the Arabidopsis Gα subunit, GPA1, that protracted handling and/or storage using the standard protocol of glycerol as a cryoprotectant are detrimental to isolating optimally functional protein (Figures 1B, 2; Supplementary Figures S1F, S3A,B). Therefore we developed a StrepII-tag expression and purification protocol that allowed rapid on-column binding to isolate highly pure protein for immediate downstream analyses (Figure 1A). The utilization of an elution buffer compatible with downstream assays, abolishing the need for buffer exchange steps, is a major advantage of the StrepII purification protocol. Even with the rapid StrepII purification protocol, our data were consistent with some loss of GPA1 activity during the purification and assay timeframe, based on the high concentrations of GPA1 required to saturate binding of 50 nM BODIPY-GTPγS, and the BODIPY signal rundown observed at lower concentrations of GPA1 (Figure 5A, compared to GNAO1 in Figure 5B). Nonetheless, matched purifications demonstrated that the loss of activity for StrepII-GPA1 was substantially less than that observed for commonly used tags: His-GPA1 or GST-GPA1 (Figure 1B). It should be noted that inclusion of GDP in Gα protein preparations is well-known to stabilize the protein (Zelent et al., 2001; Andhirka et al., 2017), as we also show for GPA1 (Figures 2A,C) and is highly advisable for several applications utilizing longer duration assays, e.g., x-ray crystallographic studies (Wall et al., 1995; Sunahara et al., 1997; Jones et al., 2011a; Liu et al., 2019). Yet, inclusion of excess GDP in elution or storage fractions is not optimal when assaying intrinsic binding affinity as excess concentrations of GDP can compete with GTP for binding to Gα (Figure 5C; Supplementary Figures S2A–C). Our rapid purification method was specifically optimized to generate protein that could be assayed prior to substantial loss of activity due to misfolding.

As our method proved to be an improvement over existing protocols for GPA1 purification (Figure 1B; Supplementary Figure S1C), we applied it to the purification of two closely related human Gα subunits, GNAI1 and GNAO1. We show our method is also applicable to human Gα subunit expression and purification such as for GNAI1 (Figure 4B). We therefore establish StrepII-mediated purification as an addition to the toolkit of possibilities for recombinant investigation of G proteins. However, His-GNAO1 outperformed StrepII-GNAO1 in our hands (Figures 4C,D), reinforcing that tag choice is not universal, and should be optimized for each protein of interest.

We then utilized our newly improved purification protocol to reexamine and further explore the following four questions of interest. 1) Does GPA1 indeed display self-activating properties? 2) Is the balance of GTP-loading of GPA1 further skewed to the active state by slow GTP hydrolysis? 3) What are the functional consequences of mutations to the serine residue important for Mg^2+^ ion coordination in the active site? 4) Given that GNAO1 displays rapid enzyme kinetics compared to GNAI1, but without the loss of stability observed in GPA1, can we employ a domain swap approach between GPA1 and GNAO1 to assess the relative contributions of the Ras vs helical domains to enzyme function and stability? As GPA1 was sensitive to differences in handling, in all assays we only directly compared proteins prepared fresh side-by-side, and we recommend that as the best practice.

### 4.1 Re-evaluation of GPA1 enzymatic kinetics

Jones et al. (2011a) characterized GPA1 as a self-activating Gα protein due to rapid GDP release followed by rapid GTP binding. They also reported slow GTP hydrolysis kinetics. Urano et al. (2012) followed this study with confirmation that Gα subunits from evolutionarily distant branches of the plant kingdom also exhibit these properties. However, both studies utilized His-tag purification protocols: Jones et al. purified Gα proteins using a 90-min batch binding step with post-elution processing steps and compared GPA1 to the slow GTP-binding Gα, GNAI1, and Urano et al. stored purified Gα subunits at −80°C with glycerol as a cryoprotectant. Though these are standard protocols for Gα purification, with hindsight we now suggest these steps are not optimal for isolation of active GPA1. It should also be noted that both studies included GDP in their elution buffers, which assists in GPA1 stabilization (Figures 2A,C) but, depending on concentration, can slow GTP binding and therefore the maximal observable hydrolysis rate (Figure 5C; Supplementary Figures S1B,C). As a result we sought to reassess the conclusions drawn from these studies by utilizing our newly developed rapid Gα purification protocol.

All of our data are consistent with the original assertion (Jones et al., 2011a) that GPA1 displays rapid GTP binding (Figures 1B,C, 5C, Supplementary Figures S1C–G, S2, S4F). Over seven independent BODIPY-GTP experiments, StrepII-GPA1 exhibited a mean association rate of 3.192 ± 0.120 (SEM) min^−1^, which matches the highest protein saturation binding rates for BODIPY-GTPγS (Supplementary Table S2) and is within the range of previously reported GPA1 association rates (Supplementary Table S5). Even when compared to GNAO1, which displays much faster binding than GNAI1 (Figure 4A), GPA1 clearly displays a faster comparative rate of GTP binding (Figures 4E,F; Supplementary Table S2). Furthermore, our analyses of GPA1 stability *in vitro* (Figures 2, 6C,D; Supplementary Figure S5D), and the inability of moderately excess concentrations of GPA1 to saturate BODIPY-GTPγS-binding (Figure 5A), suggests that this assessment of GTP binding is still an underestimate due to functional decline under assay conditions. Additionally, the new comparisons to GNAO1 presented in this report reveal the GPA1 hydrolysis rate to be less of an outlier than suggested by previous (Jones et al., 2011a) and current (Figure 4A) comparisons only to GNAI1. The apparent BODIPY-GTP net hydrolysis rate of GPA1 is only 44% lower than that of GNAO1 (Figure 4E; Supplementary Table S2) indicating relatively similar levels of activity. With regard to spontaneous nucleotide exchange, our data in Figure 5C are particularly compelling. In that GDP competition assay, GDP was provided at 10 μM, i.e., 100× in molar excess of the Gα proteins and 200× in molar excess of BODIPY-GTP. This massive overabundance of GDP was sufficient to completely outcompete BODIPY-GTP binding by GNAO1 (Figure 5C), reflecting the crucial role of GPCR-mediated stimulation in nucleotide exchange for animal Gα subunits (Dror et al., 2015; Mafi et al., 2022). Contrastingly, 10 µM GDP was only partially able to suppress GPA1 BODIPY-GTP binding activity (Figure 5C), consistent with GPA1 displaying a spontaneous nucleotide exchange activity and relatively much higher affinity for GTP than GDP, as previously reported (Johnston et al., 2007; Jones et al., 2011a; Urano et al., 2012). Overall, our data indicate that GPA1 does display rapid properties of both nucleotide exchange and GTP binding, but there likely has been underestimation of the GTP hydrolysis activity of GPA1 in the past due to choice of purification protocol. Moreover, side-by-side comparisons with two closely related mammalian Gα proteins, all isolated under optimal conditions (Figures 4A,E), reveals that the BODIPY-GTP hydrolysis rate of GPA1 falls within the range of that observed for these animal Gα subunits.

### 4.2 GNAI1^S47N^ and GNAO1^S47N^ mutants display transient GTP binding

With the advent of affordable mass patient genetic testing, a number of mutations of the equivalent sites to GNAI1^S47^/GNAO1^S47^ and GNAI1^Q204^/GNAO1^Q205^ of multiple Gα subunits have been associated with various medical conditions in ClinVar (Landrum et al., 2013) and Catalogue Of Somatic Mutations In Cancer (COSMIC) (Tate et al., 2019) databases, as summarized in Supplementary Tables S6, S7, respectively. The Q204/Q205 site resides within the G3 motif (one of five G box motifs important for nucleotide binding) of Gα subunits, mutations of which are well-known to impart a constitutively active status to Gα proteins (Kleuss et al., 1994). Mutations at this site specifically in *GNAQ* and *GNA11* are strongly linked to uveal melanoma (Van Raamsdonk et al., 2009; Silva-Rodríguez et al., 2022). The S47 site is relatively less well-understood, though it is a crucial residue within the G1 motif involved in Mg^2+^ cofactor coordination (Wall et al., 1998) and GTP binding (Slepak et al., 1993). A Gα_T_ protein, in which a region or subregions of amino acids 215–295 have been replaced with the equivalent GNAI1 residues to facilitate expression and purification, has been utilized and named Gα_T_*. When assaying binding of radiolabeled GTPγS by Gα_T_* chimeric proteins, there was an apparent discrepancy between the results of Natochin et al. (2006) who reported S43N and S43C mutants failed to bind GTP, similar to the results previously reported for an S47C mutant of GNAO1 (Slepak et al., 1993), and Ramachandran and Cerione (2011) who reported a faster rate of spontaneous GDP-GTPγS exchange for the Gα_T_*^S43N^ mutant compared to Gα_T_*. Our reassessment with real-time BODIPY-GTPγS binding suggests a mechanism by which the discrepancies evident in the above endpoint assays may be understood. The initial rates of BODIPY-GTPγS binding are faster for GNAI1^S47N^ and GNAO1^S47N^ than for the respective wild-type proteins (Figures 3C,D), while retaining Mg^2+^-dependency (Supplementary Figures S4G,H). However, the binding signal of BODIPY-GTPγS by GNAI1^S47N^ and GNAO1^S47N^ decays from an early peak, as shown by the observation that BODIPY-GTPγS signal initially increased, but then gradually decreased 3–4 min after binding initiation (Figures 3C,D). Thus, the binding signal could be missed and/or washed off if the protein is subjected to protracted handling in a radiolabeled GTPγS binding assay, and this may account for the previously reported GTP-binding discrepancy. Indeed, while Slepak et al. (1993) classified GTPγS binding by a GNAO1^S47C^ mutant as “not detected,” they did note that binding signal was “hardly detectable” above the background, yet could be quenched by unlabeled GTPγS, which would be consistent with the weak residual binding we observed at the end of our BODIPY-GTPγS assays. Our results provide additional insight into the aberrant enzymatic activities by which S47 and equivalent position mutations of human Gα subunits may manifest in disease states, although the full biochemical mechanism requires further detailed investigation. Moreover, our results illustrate an advantage of BODIPY assays in facile revelation of real-time kinetics.

### 4.3 GPA1 instability is conferred by combined effects of the Ras and helical domains, and is not inherently linked to rapid nucleotide binding

As mentioned above, studies from the Jones and Dohlman groups have indicated that the GPA1 helical domain displays both high levels of intrinsic disorder, based on comparisons of the electron density map and atomic displacement parameters of monomers determined by x-ray crystallography, and motion away from the Ras domain, as predicted by molecular dynamics simulations (Jones et al., 2011a; Jones et al., 2012). Interdomain motion is a mechanism proposed to potentiate nucleotide exchange (Rasmussen et al., 2011; Westfield et al., 2011; Dror et al., 2015; Mafi et al., 2022) and therefore these observations for GPA1 are consistent with its status as a Gα subunit capable of spontaneous nucleotide exchange (Jones et al., 2011a). As previously established, a domain substitution using the helical domain of GNAI1 conferred slower nucleotide exchange, faster GTP hydrolysis and increased stability to GPA1. Those stability experiments utilized circular dichroism over a temperature gradient of 15°C–80°C, and proteins were assayed in the presence of excess GDP. The results of Jones et al. (2011a) are therefore consistent with a coupling of high activity and low stability through the GPA1 helical domain. To further investigate this phenomenon we utilized a SYPRO Orange fluorescence assay in the presence and absence of additional nucleotides. When incubated at 25°C without additional nucleotides, GPA1 displayed reduced conformational stability (Figure 2; Supplementary Figure S5D), as expected (Zelent et al., 2001). We also observed that the enzymatic differences between GPA1 and GNAO1 were less than those between GPA1 and GNAI1 (Figure 4), though GNAO1 was likely more stable than GPA1 based on the plateau in BODIPY-GTPγS binding signal observed in Figure 5B and similarity of GNAO1 SYPRO Orange fluorescence with and without GTPγS in Supplementary Figure S5C. We speculated that the helical domain of GNAO1 may confer stability to GPA1 while also allowing the fast GTP binding kinetics of GPA1 to be retained. Indeed this proved to be the case, with GPA1^GNAO1hel^ displaying similarly rapid BODIPY-GTPγS and BODIPY-GTP binding as GPA1 (Figures 6A,B; Supplementary Table S2). When protein stability was assayed, we observed that GPA1^GNAO1hel^ displayed a similar resistance to unfolding at 25°C as GNAO1, distinguishing it from the less stable GPA1 protein (Figure 6C). When provided with a molar excess of GTPγS, GPA1 was as stable as GNAO1 and the chimeric Gα subunits (Figure 6D).

In the reciprocal domain swap, GNAO1^GPA1hel^ displayed rapid BODIPY-GTPγS binding (Figure 6A) and fast hydrolysis (Figure 6B). Unexpectedly, GNAO1^GPA1hel^ exhibited a higher basal level of SYPRO Orange interaction than the other Gα proteins, but seemingly with a relatively higher level of protein stability than GPA1 (Figures 6C,D). As the GNAO1^GPA1hel^ protein also displays strong enzymatic activity (Figures 6A,B), we conclude that the GNAO1^GPA1hel^ chimera resides in a conformation characterized by increased SYPRO accessibility, yet still functions as a molecular switch. Therefore, as the GPA1 helical domain alone was not able to confer GPA1-like instability to GNAO1, we propose that interdomain forces contribute to GPA1 instability, and that rapid kinetics and instability can be uncoupled by the use of chimeric domain swaps.

### 4.4 Future directions

Our results suggest that it will be of interest to further evaluate the GTPase activity of GPA1 in comparison to mammalian Gα subunits. Here we used BODIPY-GTP/-GTPγS to test our new, streamlined purification approach for GPA1, and screen relative G protein activities. We report our purification method as a tool for the community and highlight important contrasts to data from established methods, along with several general consistencies between our data and those of others. We also illustrate an advantage for BODIPY-GTP/GTPγS as it is a real-time method for measurement of direct binding with a sampling rate and processing speed that cannot be matched by traditional radiolabeled nucleotide approaches, as Johnston et al. previously noted (Johnston et al., 2007). These aspects are particularly useful for proteins with rapid kinetics and low stability *in vitro*. However, we also observed a drawback of the BODIPY labeling approach in the inconsistency observed between BODIPY-GTP and BODIPY-GTPγS binding for GNAI1^S47N^ and GNAO1^S47N^ (Figures 3A–D). Conjugation of a fluorophore such as BODIPY to GTP can result in differences in apparent binding compared to unlabeled GTP (Dorn et al., 2012; Goody, 2014), and therefore dictates caution when extending rate estimates to absolute rates. While our study demonstrates greater stability *in vitro* for GNAO1 than GPA1, not all human Gα subunits have been as easy to produce recombinantly as GNAO1. For example, chimeric approaches have previously been required to express Gα proteins in the soluble state, including for mammalians Gα subunits such as GNAT1 (Skiba et al., 1996; Slep et al., 2001; Ramachandran and Cerione, 2011), GNA12 and GNA13 (Kreutz et al., 2006). These chimeras integrate short regions of the kinetically particularly slow but easily purified GNAI1 enzyme. Our success with purification of the intrinsically unstable GPA1 protein indicates that our expression and rapid StrepII purification method is worth evaluating for purification of enzymatically active full length recombinant human Gα proteins that are unstable or have previously been considered difficult to produce, without the need to resort to chimeric sequence substitutions.

## Data Availability

The original contributions presented in the study are included in the article/Supplementary Material, further inquiries can be directed to the corresponding authors.

## Abbreviations

BSA, bovine serum albumin; BODIPY, boron-dipyrromethene; GAP, GTPase activating protein; GDP, guanosine diphosphate; GEF, guanine nucleotide exchange factor; GNAI1, Human guanine nucleotide-binding protein G(i) subunit alpha-1; GNAO1, Human guanine nucleotide-binding protein G(o) subunit alpha; GPA1, Arabidopsis guanine nucleotide-binding protein alpha-1 subunit; GPCR, G protein-coupled receptor) GTP, Guanosine triphosphate; GST, glutathione S-transferase; HSLB, high salt Luria-Bertani; RGS, regulator of G protein signaling; RLK, receptor-like kinase.
