# Supplementary material for: Influence of expression and purification protocols on Gα biochemical activity: kinetics of plant and mammalian G protein cycles
Source: Front Mol Biosci. 2025 Apr 7;12:1513660. doi: 10.3389/fmolb.2025.1513660 (PMC12009698; doi:10.3389/fmolb.2025.1513660)
Supplement: Supplementary file 1 [file DataSheet1.pdf]

Supporting information accompanying:

Influence of expression and purification protocols on Gα biochemical activity: kinetics of plant and mammalian G protein cycles

Timothy E. Gookin[1,2], David Chakravorty[1,2] & Sarah M. Assmann[1*]
[1] Biology Department, Pennsylvania State University, University Park, Pennsylvania 16802
[2] These authors contributed equally to the article.

ORCID IDs: 0000-0002-0994-0790 (T.E.G.); 0000-0002-6591-4853 (D.C.); 0000-0003-4541-1594 (S.M.A.).

*Authors for correspondence:
Sarah M. Assmann, sma3@psu.edu, 814-863-9579
David Chakravorty, duc16@psu.edu, 814-863-9578

This file includes:

Figure S1
Figure S2
Figure S3
Figure S4
Figure S5
Figure S6
Table S1
Table S2
Table S3
Table S4
Table S5
Table S6

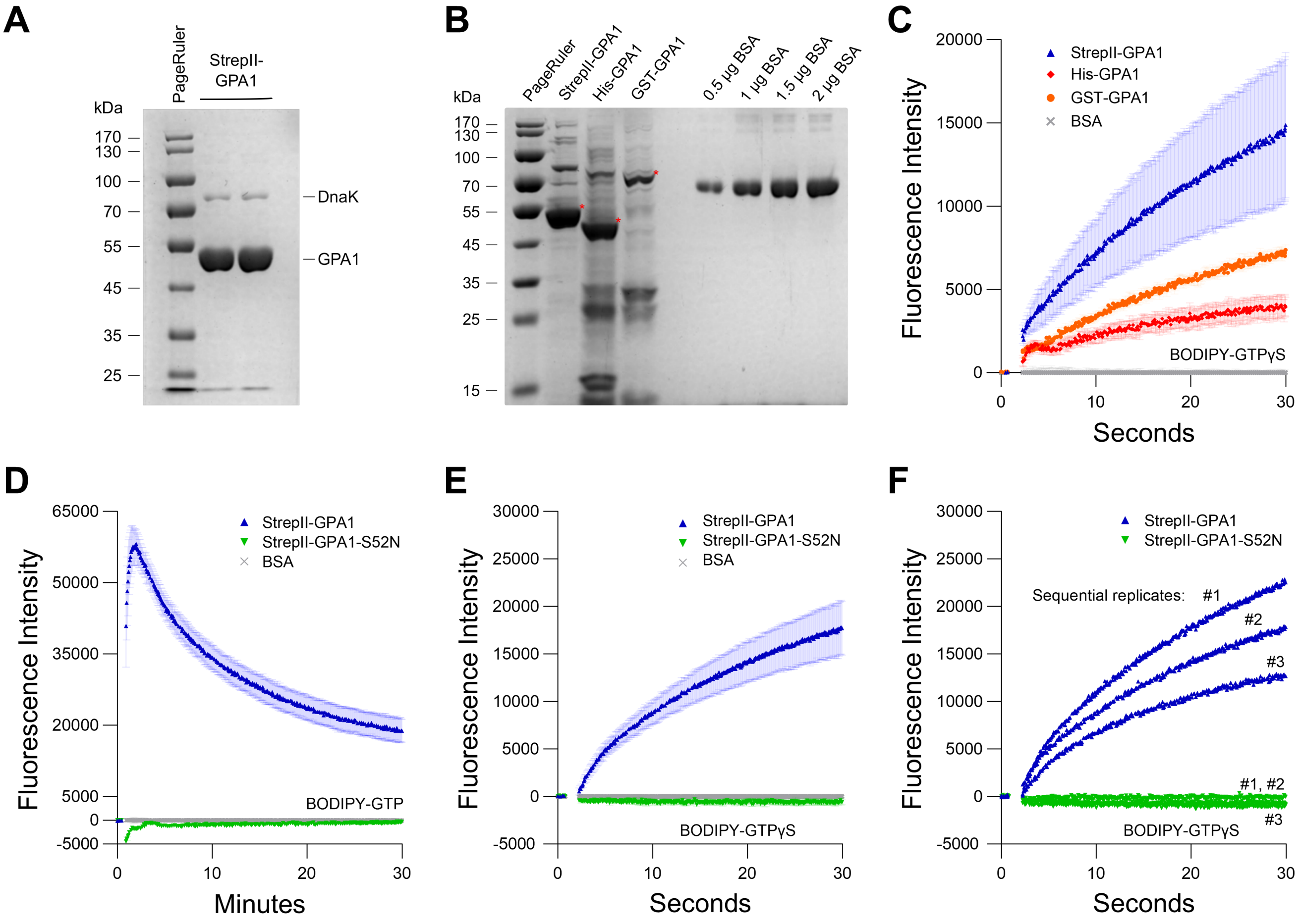

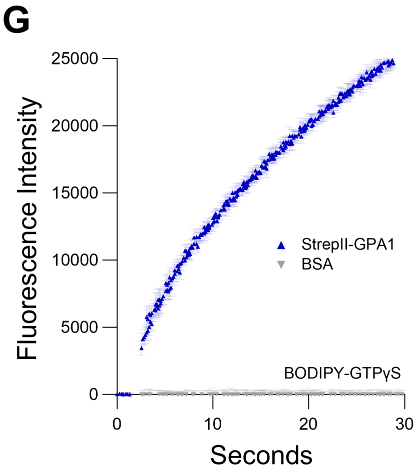

Figure S1. Comparison of GPA1 purification methods. **A.** Gel illustrating the purity of StrepII-GPA1 in our protein preparations, with the commonly co-purified ~70 kDa DnaK band. **B.** Proteins were purified in parallel for StrepII-GPA1, His-GPA1 and GST-GPA1 (marked by *) before His-GPA1 and GST-GPA1 proteins underwent buffer exchange into "EB base". Proteins were separated by SDS-PAGE for quantification of yield and qualitative assessment of purity. **C.** Comparison of the initial binding rates (note the x-axis units) of the StrepII-/His-/GST-GPA1 samples in Figure 1B when assayed with BODIPY-GTPγS. **D-E.** StrepII-GPA1^{S52N} does not display any binding activity when assayed with **D.** BODIPY-GTP or **E.** BODIPY-GTPγS. **F.** Traces of individual replicates averaged to calculate the StrepII-GPA1 data presented in panel **E**. Activity declined in order of the timing with which wells were assayed (rep 1→rep 2→rep 3), which corresponds to waiting time in the plate reader. **G.** Example of StrepII-GPA1 BODIPY-GTPγS binding assayed in well mode with tight error bars (compare with Fig. S1C). The assay presented in panel **C** was performed once as a control for the data in Figure 1B. The assays presented in panels **D** and **E** were performed eight times and the data in panel **G** five times. All replicates were performed using independently purified proteins with similar results. To generate the depicted fluorescence intensity values, the raw data replicate values were baseline-corrected using the mean replicate values for the BSA negative control, therefore the BSA traces appear near y=0, except for panel **F**, which directly presents the background subtracted replicates. The data are graphically presented as the mean ± SEM, and values below zero are not plotted for panels **C** and **F**.

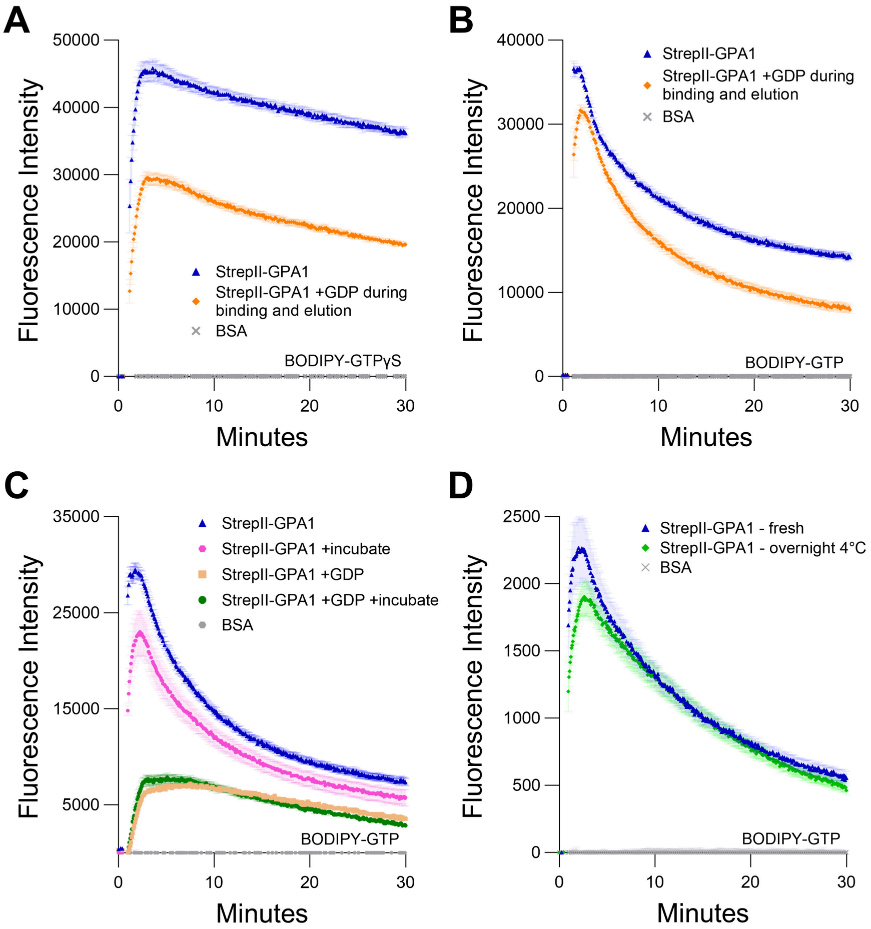

Figure S2. GDP competition with BODIPY-GTPγS and BODIPY-GTP. **A-B.** StrepII-GPA1 was purified in the presence or absence of 10 µM GDP in the binding and elution buffers. The StrepII-GPA1 sample supplemented with GDP was eluted (2.79 µM StrepII-GPA1) in the presence of 10 µM GDP. Upon dilution of StrepII-GPA1 to 100 nM final assay concentration, GDP remained in molar excess at 358 nM and competitively suppressed binding to **A.** BODIPY-GTPγS (50 nM) or **B.** BODIPY-GTP (50 nM). **C.** BODIPY-GTP binding and hydrolysis curves of StrepII-GPA1 supplemented with no GDP or 10 µM GDP and stored for 30 minutes on ice or incubated at the assay temperature of 25 °C (+incubate) prior to assay initiation. **D.** BODIPY-GTP binding and hydrolysis curves of GPA1 either freshly prepared or subjected to overnight storage at 4 °C. For the assay depicted in panel **D** the detector gain was set to 70, as opposed to 90-100 for other assays, hence the lower fluorescence intensity values. The assays presented in panels **A**, **B**, **C** and **D** were each performed twice. All replicates were performed using independently purified proteins with similar results. To generate the depicted fluorescence intensity values, the raw data values of three technical replicates for each protein were baseline-corrected using the mean replicate values for the BSA negative control and averaged, therefore the BSA traces appear near the x-axes. The data are graphically presented as the mean ± SEM and values below zero are not plotted.

A

Cryoprotectant added	Peak BODIPY-GTP fluorescence ±SEM
None	46510±1992
5% glycerol	48201±1624
10% glycerol	**49779±1370**
15% glycerol	48372±903
20% glycerol	48595±1168
25% glycerol	48845±893
5% sucrose	49907±135
8.3% sucrose	**51597±242**
10% sucrose	50026±747
15% sucrose	48236±2831
20% sucrose	46618±3872
25% sucrose	44786±4538

B

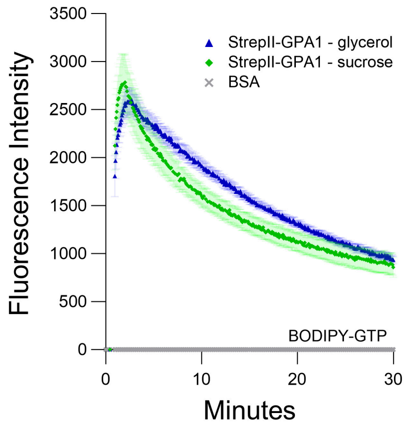

Figure S3. Activity of StrepII-GPA1. **A.** Peak fluorescence values of BODIPY-GTP curves of StrepII-GPA1 supplemented with the indicated concentrations of the cryoprotectants glycerol or sucrose and stored at -80 °C for 3 weeks. **B.** Comparison of BODIPY-GTP binding and hydrolysis activities of StrepII-GPA1 stored at -80 °C for 3 weeks with either 10% glycerol or 8.33% sucrose added to the elution fraction (which contains 5% glycerol) as a cryoprotectant. For the assay depicted in panel **B** the detector gain was set to 70, as opposed to 90-100 for other assays, hence the lower fluorescence intensity values. The assay presented in panel **A** was performed once, with three technical replicates, while the assay presented in panel **B** was performed twice, using independently purified proteins with similar results. To generate the depicted fluorescence intensity values, the raw data values of three technical replicates for each protein were baseline-corrected using the mean replicate values for the BSA negative control and averaged, therefore the BSA traces appear near the x-axes in panel **B**. The data are graphically presented as the mean ± SEM and values below zero are not plotted.

A

```
>GNAI1ch
ATGGGCTGCACCCTGTCTGCCGAAGATAAAGCGGCAGTTGAACGCTCCAAAATGATCGACCGCAACCTGCGTGAAGACGGTGAAAAAGCCGCGCGTGAAGTTAAACTGCTCCTGCTCGGTGC
AGGCGAATCTGGCAAATCCACCATCGTTAAACAGATGAAAATCATCCACGAAGCCGGTTATTCTGAAGAGGAATGTAAACAGTACAAAGCAGTGGTTTACAGCAACACCATCCAGTCTATTA
TCGCTATCATTCGTGCGATGGGTCGTCTGAAAATCGACTTTGGTGACAGCGCCCGTGCGGATGACGCACGCCAGCTGTTTGTGCTGGCAGGTGCAGCGGAGGAAGGCTTTATGACTGCAGAA
CTGGCCGGTGTTATCAAACGTCTGTGGAAAGATTCTGGTGTACAGGCCTGTTTCAACCGTTCCCGTGAATACCAGCTGAACGATTCTGCAGCGTACTATCTGAACGACCTGGACCGTATCGC
TCAGCCGAACTACATCCCGACTCAGCAGGATGTTCTGCGTACTCGTGTGAAAACTACCGGTATTGTTGAAACCCACTTTACTTTCAAAGATCTGCACTTTAAAATGTTTGATGTGGGTGGCC
AGCGTTCTGAACGTAAGAAATGGATTCACTGCTTCGAAGGTGTGACCGCGATTATCTTCTGTGTAGCACTGTCTGACTACGACCTGGTTCTGGCCGAAGATGAGGAAATGAACCGTATGCAC
GAAAGCATGAAACTGTTTGACAGCATCTGTAATAACAAATGGTTTACCGATACCAGCATTATCCTGTTTCTGAACAAAAAAGATCTGTTTGAGGAAAAAATCAAGAAAAGCCCGCTGACCAT
CTGCTACCCGGAATATGCGGGTTCTAACACCTATGAAGAGGCGGCTGCATATATTCAGTGTCAGTTTGAAGACCTGAACAAACGTAAAGACACCAAAGAAATCTACACCCACTTCACCTGTG
CCACCGATACTAAAAACGTGCAGTTTGTTTTTGATGCTGTAACCGATGTGATCATTAAAAATAACCTGAAGGATTGCGGTCTGTTCTAA
```

B

```
GNAI1wt     MGCTLSAEDKAAVERSKMIDRNLREDGEKAAREVKLLLLGAGESGKSTIVKQMKIIHEAG   60
GNAI1ch     MGCTLSAEDKAAVERSKMIDRNLREDGEKAAREVKLLLLGAGESGKSTIVKQMKIIHEAG   60
            ************************************************************

GNAI1wt     YSEEECKQYKAVVYSNTIQSIIAIIRAMGRLKIDFGDSARADDARQLFVLAGAAEEGFMT   120
GNAI1ch     YSEEECKQYKAVVYSNTIQSIIAIIRAMGRLKIDFGDSARADDARQLFVLAGAAEEGFMT   120
            ************************************************************

GNAI1wt     AELAGVIKRLWKDSGVQACFNRSREYQLNDSAAYYLNDLDRIAQPNYIPTQQDVLRTRVK   180
GNAI1ch     AELAGVIKRLWKDSGVQACFNRSREYQLNDSAAYYLNDLDRIAQPNYIPTQQDVLRTRVK   180
            ************************************************************

GNAI1wt     TTGIVETHFTFKDLHFKMFDVGGQRSERKKWIHCFEGVTAIIFCVALSDYDLVLAEDEEM   240
GNAI1ch     TTGIVETHFTFKDLHFKMFDVGGQRSERKKWIHCFEGVTAIIFCVALSDYDLVLAEDEEM   240
            ************************************************************

GNAI1wt     NRMHESMKLFDSICNNKWFTDTSIILFLNKKDLFEEKIKKSPLTICYPEYAGSNTYEEAA   300
GNAI1ch     NRMHESMKLFDSICNNKWFTDTSIILFLNKKDLFEEKIKKSPLTICYPEYAGSNTYEEAA   300
            ************************************************************

GNAI1wt     AYIQCQFEDLNKRKDTKEIYTHFTCATDTKNVQFVFDAVTDVIIKNNLKDCGLF*   354
GNAI1ch     AYIQCQFEDLNKRKDTKEIYTHFTCATDTKNVQFVFDAVTDVIIKNNLKDCGLF*   354
            ******************************************************
```

C

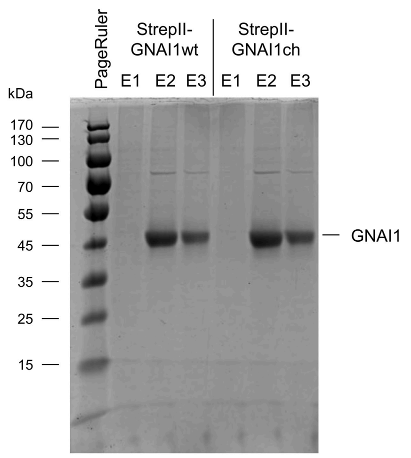

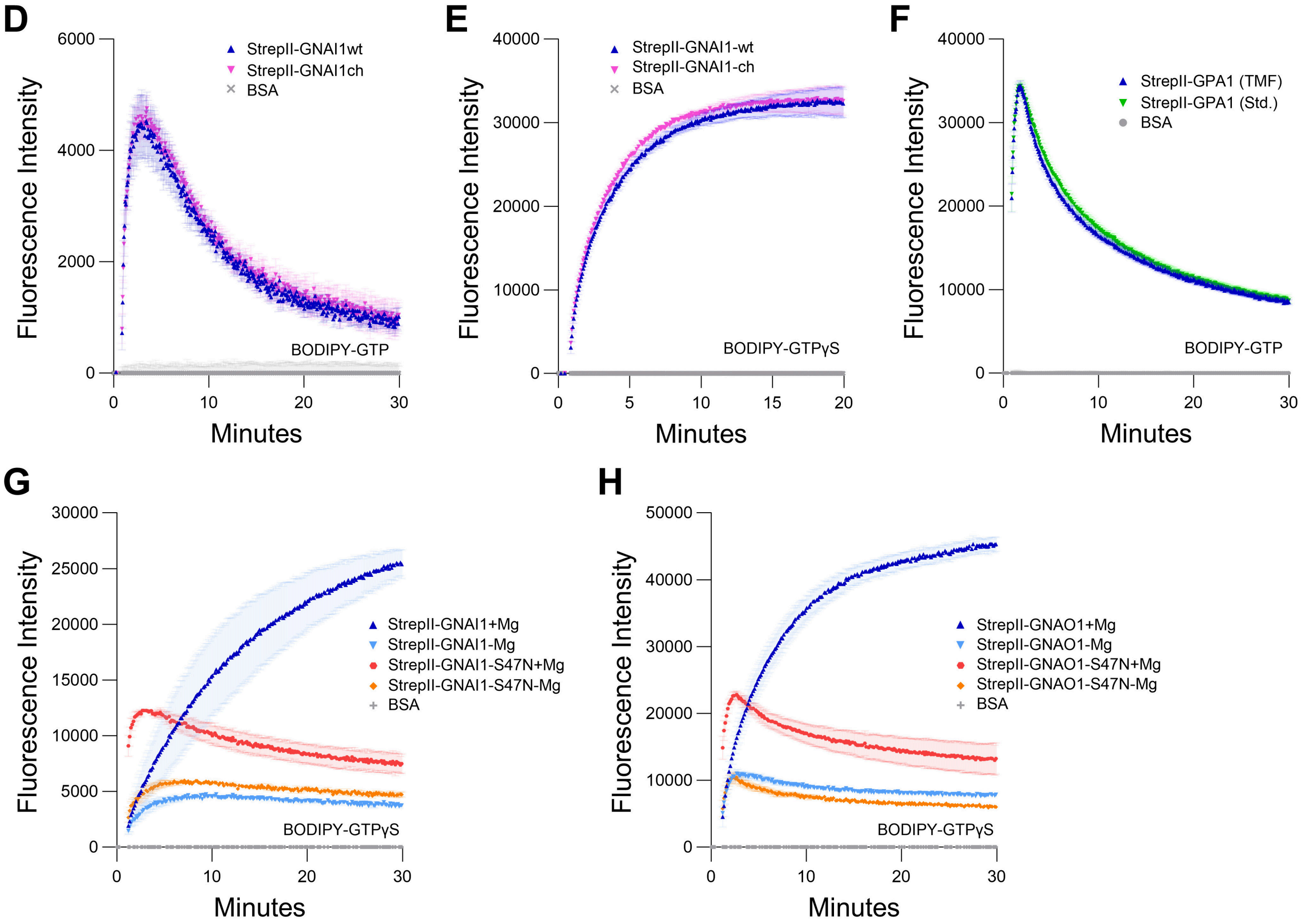

Figure S4. Control data for GNAI1 codon harmonization, and buffer reagent choices. **A.** DNA sequence of the codon harmonized GNAI1ch synthesized clone. **B.** Alignment of GNA1wt (native) and GNAI1ch protein sequences, generated with Clustal Omega. **C.** SDS-PAGE illustrates the similar relative yield and purity of Strep-tag purified GNAI1wt and GNAI1ch proteins. **D-E.** Similar activities of StrepII-GNAI1wt vs. StrepII-GNAI1ch (250 nM each) for **D.** BODIPY-GTP binding and hydrolysis, and **E.** BODIPY-GTPγS binding. Note the 20-minute x-axis scale. **F.** Comparison of the BODIPY-GTP binding and hydrolysis activities of 100 nM StrepII-GPA1 purified and assayed in buffers prepared with standard (Std.) grade reagents or trace metal free (TMF) grade reagents. **G-H.** Mg^{2+} dependency for BODIPY-GTPγS binding by **G.** 200 nM GNAI1 vs. 200 nM $GNAI1^{S47N}$ or **H.** 100 nM GNAO1 vs. 100 nM $GNAO1^{S47N}$. The assays presented in panels **D**, **E** and **F** were performed once and the assays in panels **G** and **H** were performed twice using independently purified proteins with similar results. To generate the depicted fluorescence intensity values, the raw data values of three (panels **G** and **H**) or four (panels **D-F**) technical replicates for each protein were baseline-corrected using the mean replicate values for the BSA negative control and averaged, therefore the BSA traces appear near the x-axes. The data are graphically presented as the mean ± SEM and values below zero are not plotted.

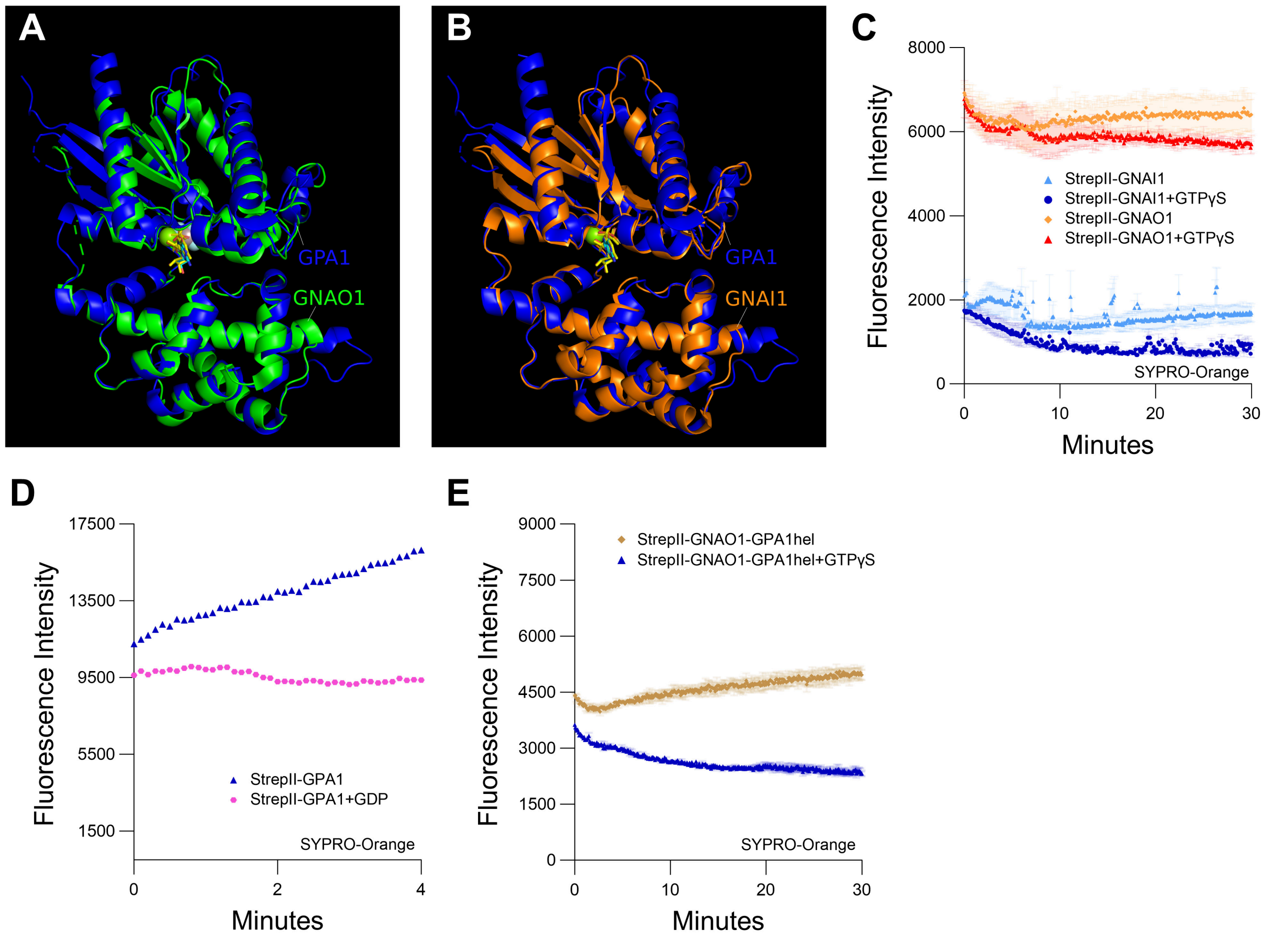

Figure S5. A-B. Structural alignments of empirically derived **A.** GPA1-GNAO1 and **B.** GPA1-GNAI1 structures. PDB structure 2XTZ chain A (GPA1 – blue) was aligned in PyMol with 3C7K chain A (GNAO1 – green) or 1GIA chain A (GNAI1 – orange). The nucleotide (yellow for GPA1/light and dark blue for GNAO1 and GNAI1, respectively) is located in the binding pocket within the interdomain cleft, which is flanked by the Ras domain (upper domain) and helical domain (lower domain) in both panels. **C.** SYPRO Orange protein unfolding assay in the presence or absence of GTPγS for 600 nM StrepII-GNAI1 or StrepII-GNAO1. Note the reduced magnitude of fluorescence intensity values compared to StrepII-GPA1 in Figures 2A and 2B. The initial decrease in SYPRO Orange signal may result from temperature equilibration, as reactions were loaded immediately following sample preparation on ice. **D.** SYPRO Orange assay of individual wells without replication to allow for rapid setup demonstrates that unfolding of GPA1 (600 nM) in vitro when not provided with excess nucleotide is almost immediate. **E.** An example of a GNAO1^{GPA1hel} SYPRO Orange protein unfolding assay in which the rapid signal variation between timepoints displayed in Figures 6C and 6D was not observed, indicating that this variation is not an inherent property of the protein. (Note the same y-axis scale was used in Figures 6C, 6D and S5E.) The assays presented in panels **C**, **D** and **E** were performed once, as additional evidence to complement main text Figures 2-5, 6C, and 6C-D, respectively. The data in panels **C** and **E** are graphically presented as the mean ± SEM of two technical replicates, while the data in panel **D** represent single measurements.

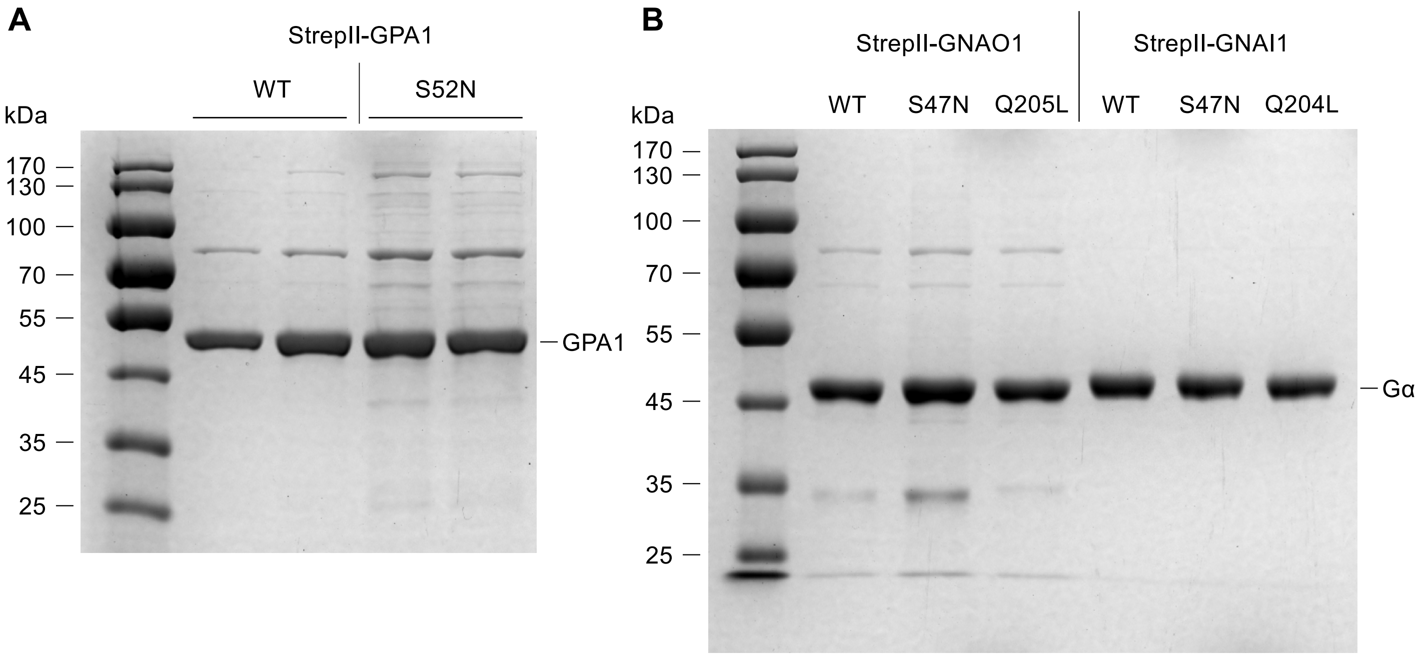

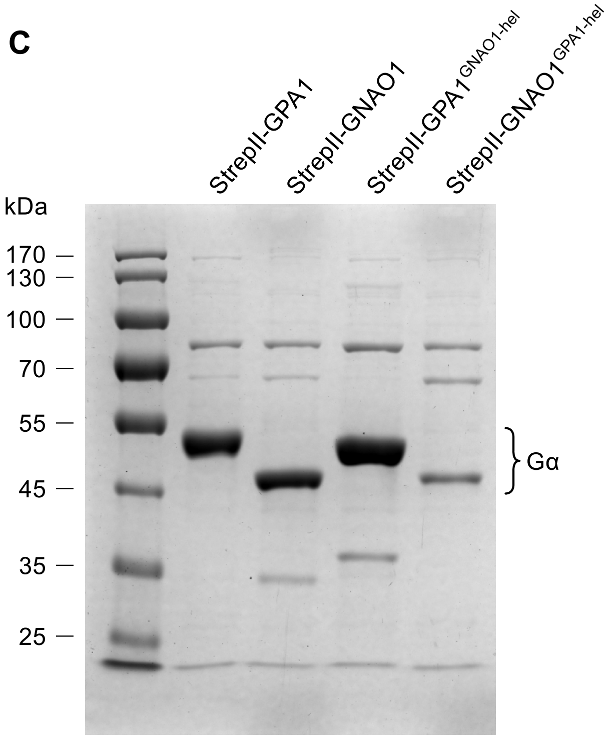

Figure S6. Point mutations and domain swaps did not affect the integrity of StrepII-purified Gα subunits. **A.** SDS-PAGE comparing the wild-type StrepII-GPA1 protein to the StrepII-GPA1^{S52N} mutant. Ten µl of StrepII-GPA1 E2 fractions were compared to 20 µl of StrepII-GPA1^{S52N} E2 fractions due to the lower yield of the mutant protein. Note the increased volume loaded for the mutant protein is likely responsible for the increase in background bands evident in the StrepII-GPA1^{S52N} lanes. **B.** Gel electrophoresis of 20 µl of E2 fractions of StrepII-GNAO1 and 10 µl of E2 fractions of StrepII-GNAI1 proteins assayed in Figure 3. **C.** Gel electrophoresis of GPA1-GNAO1 helical domain swap proteins assayed in Figure 6. Ten µl of the E2 fraction of StrepII-GPA1 and 20 µl of E2 fractions of all other proteins were separated on the gel.

Table S1. Sequences of primers used in this study.

**Primer**	**Sequence**	**Use**
GPA1f-NcoI	GGGCCATGGGCTTACTCTGCAG	Cloning *GPA1* into pSTTa
GPA1r-BspEI	GGGTCCGGATCATAAAAGGCCAGCCTCCAG	
GPA1f-BspHI	GCGTCATGATGGGCTTACTCTGCAG	Cloning the *GPA1*^{GNAO1hel} construct into pSTTa
GNAI1f-NcoI	GCGCCATGGGCTGCACCCTGTC	Cloning codon harmonized *GNAI1* into pSTTa
GNAI1r-BspEI	GGGTCCGGATTAGAACAGACCGCAATCCTTC	
GNAI1f-wt-NcoI	GCACCATGGGCTGCACGCTGAG	Cloning the native (wild-type) *GNAI1* into pSTTa
GPA1-37f-NcoI	GCGCCATGGGTCATATTCGGAAGCTTTTGCTAC	
GNAO1f-BspHI	GCGTCATGATGGGATGTACTCTGAGCG	Cloning *GNAO1* into pSTTa
GNAO1r	TCAGTACAAGCCGCAGCCC	
GPA1S52NF	CTGGAAAAAAATACAATTTTTAAGC	S52N mutagenesis of *GPA1*
GPA1S52NR	GCTTAAAAATTGTATTTTTTCCAG	
GNAI1-S47N-rf	GGCAAAAACACCATCGTTAAACAGATGAAAATCATCC	S47N mutagenesis of *GNAI1*
GNAI1-S47N-rr	ACGATGGTGTTTTTGCCAGATTCGCCTGC	
GNAI1-Q204L-rf	GGGTGGCCTGCGTTCTGAACGTAAGAAATGG	Q204L mutagenesis of *GNAI1*
GNAI1-Q204L-rr	CAGAACGCAGGCCACCCACATCAAACAT	
GNAO1-S47N-rf	AGGAAAAAAACACCATTGTGAAGCAGATGAAG	S47N mutagenesis of *GNAO1*
GNAO1-S47N-rr	CAATGGTGTTTTTTCCTGATTCTCCAGC	
GNAO1-Q205L-rf	CGGAGGCCTGCGATCTGAACGCAAGAAG	Q205L mutagenesis of *GNAO1*
GNAO1-Q205L-rr	CAGATCGCAGGCCTCCGACGTCAAACAG	
GPA1-GNAO1hel-or	GTACTGTTTCACGTCTTCTCCATCAAATCCCGTTTGGAATAGAAG	Reverse primer of the N-terminal fragment of the *GPA1* Ras domain (for overlap extension PCR)
GPA1-GNAO1hel-of	GAGCAGGACATCCTCCGAGCAAGAGTTCGCACAACTG	Forward primer of the C-terminal fragment of the *GPA1* Ras domain (for overlap extension PCR)
GPA1hel-f2	GAAGGAGAACTAAAGAGCTATGTTC	Forward primer of the *GPA1* helical domain (for overlap extension PCR)
GPA1hel-r	ATAAAGTACATCCTCCTTAGTTGG	Reverse primer of the *GPA1* helical domain (for overlap extension PCR)
GNAO1-GPA1hel-or	GAACATAGCTCTTTAGTTCTCCTTCGGAGAAGCCATCTTCATGGATG	Reverse primer of the N-terminal fragment of the

		GNAO1 Ras domain (for overlap extension PCR)
GNAO1-GPA1hel-of	CCAACTAAGGAGGATGTACTTTATACCAGGGTCAAAACCACTG	Forward primer of the C-terminal fragment of the *GNAO1* Ras domain (for overlap extension PCR)
GNAO1hel-f	GGAGAAGACGTGAAACAGTACAAG	Forward primer of the *GNAO1* helical domain (for overlap extension PCR)
GNAO1hel-r	TCGGAGGATGTCCTGCTC	Reverse primer of the *GNAO1* helical domain (for overlap extension PCR)
GPA1f	ATGGGCTTACTCTGCAGTA	Cloning *GPA1* into pCR8
GPA1rs	TCATAAAAGGCCAGCCTCC	
GNAI1f	ATGGGCTGCACCCTGTCTG	Cloning codon harmonized *GNAI1* into pCR8
GNAI1r	TTAGAACAGACCGCAATCCTTCAG	
GNAO1f	ATGGGATGTACTCTGAGCG	Cloning *GNAO1* into pCR8
GNAO1r	TCAGTACAAGCCGCAGCCC	
RGS1 NcoI 763F	GGATCCATGGGCTCGAGTAACCAACCTCTTCTTTCAC	Cloning the region corresponding to the C-terminal cytosolic RGS domain of *RGS1* into pSTTa
RGS1 BspEI 1355Rs	TCAAGCTCCGGATTAACCGGGACTACTGCATCTGGAAC	

Table S2. Summary of calculated binding (BODIPY-GTPγS), binding and hydrolysis (BODIPY-GTP), and signal decay (SYPRO Orange) rates calculated in curve fitting analyses performed on the traces found within this manuscript.

		Observed association			Observed decay			
		k (min^{-1})	R^2	k as %	k (min^{-1})	R^2	k as %	
**Fig. 1B**	StrepII-GPA1	3.139	0.980	100.0	0.09426	0.9988	100	The average observed BODIPY-GTP
BODIPY-GTP	His-GPA1	1.216	0.970	38.7	0.01391	0.9975	14.8	association rate for Strep-GPA1 over
	GST-GPA1	2.415	0.991	76.9	0.0575	0.9984	61	seven figures = 3.192 ± 0.120 (SEM) min^{-1}
**Fig. 2D**	GTPγS T=30 min	N.D.	N.D.	N.D.	0.077	0.988	100	
SYPRO-Orange	GTPγS T=20 min	N.D.	N.D.	N.D.	0.113	0.984	146.9	
	GTPγS T=15 min	N.D.	N.D.	N.D.	0.185	0.989	239.6	
	GTPγS T=10 min	N.D.	N.D.	N.D.	0.291	0.975	378.0	
	GTPγS T=5 min	N.D.	N.D.	N.D.	0.682	0.964	885.0	
**Fig. 4A**	StrepII-GPA1	3.156	0.955	100.0	0.1395	0.9908	100.0	
BODIPY-GTP	StrepII-GNAO1	1.065	0.999	33.7	0.1512	0.9975	108.4	
	StrepII-GNAI1	0.844	0.707	26.7	0.1094	0.9602	78.4	
**Fig. 4C**	StrepII-GNAO1	0.671	0.968	100.0	0.09089	0.9982	100.0	
BODIPY-GTP	His-GNAO1	1.220	0.958	181.8	0.2629	0.9982	289.3	
**Fig. 4E**	StrepII-GPA1	3.251	0.823	100.0	0.1127	0.9975	100.0	
BODIPY-GTP	His-GNAO1	0.801	0.968	24.6	0.2016	0.9976	178.9	
**Fig. 4F**	StrepII-GPA1	1.442	0.977	100.0	N.D.	N.D.	N.D.	
BODIPY-GTPγS	His-GNAO1	0.206	0.999	14.3	N.D.	N.D.	N.D.	
**Fig. 5A**	**StrepII-GPA1**							
BODIPY-GTPγS	1.2 μM	3.531	0.9278	N.D.	N.D.	N.D.	N.D.	Our curve fitting approach for BODIPY-GTP is
	800 nM	3.364	0.9326	N.D.	N.D.	N.D.	N.D.	validated by the protein saturation binding
	400 nM	2.894	0.9209	N.D.	N.D.	N.D.	N.D.	kinetic values obtained using BODIPY-GTPγS.
	200 nM	1.946	0.7887	N.D.	N.D.	N.D.	N.D.	
	100 nM	1.592	0.253	N.D.	N.D.	N.D.	N.D.	
**Fig. 5B**	**His-GNAO1**							
BODIPY-GTPγS	1.2 μM	1.805	0.973	N.D.	N.D.	N.D.	N.D.	
	800 nM	1.461	0.9667	N.D.	N.D.	N.D.	N.D.	
	400 nM	0.9524	0.9737	N.D.	N.D.	N.D.	N.D.	
	200 nM	0.4934	0.9916	N.D.	N.D.	N.D.	N.D.	
	100 nM	0.2798	0.9978	N.D.	N.D.	N.D.	N.D.	
**Fig. 6A**	StrepII-GPA1	1.126	0.965	100.0	N.D.	N.D.	N.D.	
BODIPY-GTPγS	StrepII-GPA1-GNAO1hel	0.533	0.996	47.4	N.D.	N.D.	N.D.	
	StrepII-GNAO1	0.134	0.999	11.9	N.D.	N.D.	N.D.	
	StrepII-GNAO1-GPA1hel	1.608	0.955	142.8	N.D.	N.D.	N.D.	
**Fig. 6B**	StrepII-GPA1	3.088	0.972	100.0	0.144	0.992	100	
BODIPY-GTP	StrepII-GPA1-GNAO1hel	0.867	0.911	28.1	0.116	0.999	80.7	
	StrepII-GNAO1	0.806	0.900	26.1	0.117	0.996	81.4	
	StrepII-GNAO1-GPA1hel	2.651	0.952	85.8	0.505	0.993	351.5	
**Fig. S2A**	StrepII-GPA1	1.478	0.973	100.0	N.D.	N.D.	N.D.	
BODIPY-GTPγS	StrepII-GPA1 with GDP-binding/elution	1.137	0.964	76.9	N.D.	N.D.	N.D.	
**Fig. S2B**	StrepII-GPA1	3.356	0.945	100.0	0.137	0.991	100.0	
BODIPY-GTP	StrepII-GPA1 with GDP-binding/elution	2.272	0.9803	67.7	0.136	0.997	100.5	
**Fig. S2C**	StrepII-GPA1	3.706	0.965	100.0	0.136	0.997	100.0	
BODIPY-GTP	StrepII-GPA1+GDP	0.718	0.923	19.4	0.018	0.993	12.9	
	StrepII-GPA1+incubate	2.123	0.983	57.3	0.121	0.997	89.3	
	StrepII-GPA1+GDP+incubate	0.819	0.909	22.1	0.034	0.997	25.2	
**Fig. S2D**	StrepII-GPA1 - fresh	2.648	0.989	100.0	0.091	0.998	100.0	
BODIPY-GTP	StrepII-GPA1 - overnight 4°C	1.814	0.994	68.5	0.055	0.999	60.1	
**Fig. S3B**	StrepII-GPA1 - sucrose	3.032	0.9786	100	0.104	0.9958	100	
BODIPY-GTP	StrepII-GPA1 - glycerol	2.339	0.9896	77.1	0.047	0.9993	44.8	

Table S3. Yield comparison of StrepII-GPA1, His-GPA1 and GST-GPA1 when proteins were purified from 600 ml induction cultures side-by-side on five different days. Yields are presented as nmol (µg).

	Set 1	Set 2	Set 3	Set 4	Set 5	Average
StrepII-GPA1	0.67 (33.6)	0.74 (37.0)	0.96 (47.9)	1.09 (54.7)	1.20 (60.0)	0.93 (46.6)
His-GPA1	0.46 (22.1)	0.56 (25.9)	0.37 (17.4)	0.53 (25.3)	0.65 (31.1)	0.51 (24.4)
GST-GPA1	0.11 (7.9)	0.10 (6.9)	0.13 (9.5)	0.14 (10.2)	0.11 (7.7)	0.12 (8.4)

Table S4. Maximal amplitudes of background-corrected BODIPY-GTP and BODIPY-GTPγS fluorescence signals derived from the data presented in this manuscript

		**Analyzed proteins**					
**Figure**	**Statistic**	StrepII-GPA1	His-GPA1	GST-GPA1	BSA		
1B	Maximum	43368	23353	32353	1E-12		
		StrepII-GPA1	StrepII-GPA1 + StrepII-RGS1c (1:2)	StrepII-GPA1 + StrepII-RGS1c (1:4)			
1C	Maximum	17868	15806	12007			
		StrepII-GPA1	StrepII-GPA1+GDP	Buffer			
2A	Maximum	27639	15902	0			
		StrepII-GPA1	StrepII-GPA1+GTPγS	Buffer			
2B	Maximum	21683	9736	0			
		StrepII-GNAI1	StrepII-GNAI1-S47N	StrepII-GNAI1-Q204L	BSA		
3A	Maximum	2577	706	7406	6E-13		
		StrepII-GNAO1	StrepII-GNAO1-S47N	StrepII-GNAO1-Q205L	BSA		
3B	Maximum	14435	867	6236	6E-13		
		StrepII-GNAI1	StrepII-GNAI1-S47N	StrepII-GNAI1-Q204L	BSA		
3C	Maximum	26196	12680	18212	6E-13		
		StrepII-GNAO1	StrepII-GNAO1-S47N	StrepII-GNAO1-Q205L	BSA		
3D	Maximum	45862	23743	12356	6E-13		
		StrepII-GPA1	StrepII-GNAO1	StrepII-GNAI1	BSA		
4A	Maximum	42897	19055	2555	1E-12		
		StrepII-GNAI1	His-GNAI1	BSA			
4B	Maximum	3063	3942	1E-12			
		StrepII-GNAO1	His-GNAO1	BSA			
4C	Maximum	9231	33077	6E-13			
		StrepII-GNAO1	His-GNAO1	BSA			
4D	Maximum	24332	29422	3E-13			
		StrepII-GPA1	His-GNAO1	BSA			
4E	Maximum	23300	14629	6E-13			
		StrepII-GPA1	His-GNAO1	BSA			
4F	Maximum	21945	51177	1E-12			5A and 5B share the BSA sample
		100 nM GPA1	200 nM GPA1	400 nM GPA1	800 nM GPA1	1.2 µM GPA1	BSA
5A	Maximum	5179	9592	12331	13111	13181	0
		100 nM GNAO1	200 nM GNAO1	400 nM GNAO1	800 nM GNAO1	1.2 µM GNAO1	BSA
5B	Maximum	10292	10711	10652	10613	10593	0
		StrepII-GPA1+GDP	StrepII-GPA1	His-GNAO1+GDP	His-GNAO1	BSA	
5C	Maximum	8223	23632	-86	12233	6E-13	
		StrepII-GPA1	StrepII-GPA1-GNAO1hel	StrepII-GNAO1	StrepII-GNAO1-GPA1hel	BSA	
6A	Maximum	39991	50383	50320	64860	6E-13	
		StrepII-GPA1	StrepII-GPA1-GNAO1hel	StrepII-GNAO1	StrepII-GNAO1-GPA1hel	BSA	
6B	Maximum	32316	44123	11572	11059	6E-13	
		StrepII-GPA1	His-GPA1	GST-GPA1	BSA		
S1C	Maximum	15211	4317	7662	0		
		StrepII-GPA1	StrepII-GPA1-S52N	BSA			
S1D	Maximum	58194	34	1E-12			
		StrepII-GPA1	StrepII-GPA1-S52N	BSA			
S1E	Maximum	18506	109	1E-12			
		StrepII-GPA1 (Rep #1)	StrepII-GPA1 (Rep #2)	StrepII-GPA1 (Rep #3)	StrepII-GPA1-S52N (Rep #1)	StrepII-GPA1-S52N (Rep #2)	StrepII-GPA1-S52N (Rep #3)
S1F	Maximum	23570	18638	13389	379	161	81
		StrepII-GPA1	BSA				
S1G	Maximum	25763	2E-12				
		StrepII-GPA1	StrepII-GPA1 +GDP-binding/elution	BSA			
S2A	Maximum	45839	29542	6E-13			
		StrepII-GPA1	StrepII-GPA1 GDP-binding/elution	BSA			
S2B	Maximum	36645	31655	6E-13			
		StrepII-GPA1	StrepII-GPA1 +GDP	StrepII-GPA1 +incubate	StrepII-GPA1 +GDP +incubate	BSA	
S2C	Maximum	29485	7212	23011	7833	6E-13	
		StrepII-GPA1 - fresh	StrepII-GPA1 - overnight 4°C	BSA			
S2D	Maximum	2269	1904	0			
		StrepII-GPA1 - sucrose	StrepII-GPA1 - glycerol	BSA			
S3B	Maximum	2786	2605	0			
		StrepII-GNAI1wt	StrepII-GNAI1ch	BSA			
S4D	Maximum	4527	4756	0			
		StrepII-GNAI1-wt	StrepII-GNAI1-ch	BSA			
S4E	Maximum	32761	32948	0			
		StrepII-GPA1 (TMF)	StrepII-GPA1 (Std.)	BSA			
S4F	Maximum	34352	34438	0			
		StrepII-GNAI1+Mg	StrepII-GNAI1-Mg	StrepII-GNAI1-S47N+Mg	StrepII-GNAI1-S47N-Mg	BSA	
S4G	Maximum	25918	4754	12264	5994	6E-13	
		StrepII-GNAO1+Mg	StrepII-GNAO1-Mg	StrepII-GNAO1-S47N+Mg	StrepII-GNAO1-S47N-Mg	BSA	
S4H	Maximum	45718	11022	22797	10563	6E-13	
		StrepII-GPA1	StrepII-GPA1+GDP				
S5D	Maximum	16143	9371				
		StrepII-GNAO1-GPA1hel	StrepII-GNAO1-GPA1hel+GTPγS				
S5E	Maximum	5055	3623				

Table S5. Previously reported GPA1 GTP association rates in the literature.

**Calculated GTP association rates of GPA1**	**Method**	**Study**
1.44 min^{-1}	[^{35}S]GTPγS binding	Johnston et al., 2007
14.4 min^{-1}	BODIPY-GTPγS binding	Johnston et al., 2007
1.7 min^{-1}	Intrinsic tryptophan fluorescence	Jones et al., 2011a
3.4 min^{-1}	MANT-GMPPNP binding	Jones et al., 2011b
4.1 min^{-1}	Intrinsic tryptophan fluorescence	Jones et al., 2011b
5.8 min^{-1}	[^{35}S]GTPγS binding	Urano et al., 2012
3.63 min^{-1}	Intrinsic tryptophan fluorescence	Urano et al., 2012
2.6 min^{-1}	Intrinsic tryptophan fluorescence	Jones et al., 2012
0.9 min^{-1}	[35S]GTPγS binding	Jones et al., 2012

Table S6. Clinvar data associated with equivalent sites to GNAI1^{S47}/GNAO1^{S47} and GNAI1^{Q204}/GNAO1^{Q205} of Gα subunits.

**Gene**	**Mutations to protein**	**Conditions**
GNAI3	S47R	Auriculocondylar syndrome 1
GNAI3	S47N	Auriculocondylar syndrome 1
GNAO1	S47N	Developmental and epileptic encephalopathy 17, early infantile epileptic encephalopathy with suppression bursts
GNAT2	S47G	Achromatopsia 4
GNAT3	S47T	Inborn genetic diseases
GNAI1	Q204R	Neurodevelopmental disorder with hypotonia, impaired speech, and behavioral abnormalities
GNAO1	Q205P	Developmental and epileptic encephalopathy 17
GNAO1	Q205L	Early infantile epileptic encephalopathy with suppression bursts
GNAT1	Q200E	Congenital stationary night blindness autosomal dominant 3
GNAS	Q227K, Q227H	McCune-Albright syndrome
GNAS	Q227L	Neoplasm
GNAS	Q227R, Q227H	Pituitary adenoma 3, multiple types
GNAQ	Q209H	Familial multiple nevi flammei
GNAQ	Q209L, Q209P	Melanoma, uveal melanoma

GNAQ	Q209R	Melanoma, abnormality of cardiovascular system morphology, Sturge-Weber syndrome
GNA11	Q209R, Q209E	Not provided
GNA11	Q209P, Q209L	Melanoma, uveal melanoma
GNA14	Q205L	Congenital tufted angioma, Kaposiform hemangioendothelioma, Pyogenic granuloma

Table S7. COSMIC data associated with equivalent sites to GNAI1^{S47}/GNAO1^{S47} and GNAI1^{Q204}/GNAO1^{Q205} of Gα subunits.

**Gene**	**Mutations to protein**	**Conditions**	**Samples**
GNAI2	S47N	Lymphoid neoplasm, large intestine carcinoma	7
GNAT1	S43N	Large intestine carcinoma	1
GNAS	S54R	Malignant melanoma	1
GNAL	S56I	Liver neoplasm	1
GNA11	S53G	Large intestine carcinoma	3
GNA13	S62P	Burkitt lymphoma	3
GNA15	S56R	Ewings sarcoma-peripheral primitive neuroectodermal tumour	1
GNAI3	Q204R	Cervical carcinoma, Malignant melanoma	2
GNAI3	Q204E	Malignant melanoma	1
GNAS	Q227L	Malignant melanoma, lung, pancreatic, liver and salivary gland carcinomas, pituitary and stomach adenomas, fibrous dysplasia	47
GNAS	Q227R	Biliary tract, small intestine, large intestine, lung and pancreatic carcinomas, pituitary, stomach, thyroid (Adenoma-nodule-goitre) and pituitary adenomas	25

GNAS	Q227H	Adrenal gland, lung, prostate, small intestine and thyroid carcinomas, small intestine and pituitary adenomas, Adenoma-nodule-goitre (thyroid), Haematopoietic and pancreatic neoplasms, Pseudomyxoma peritonei	21
GNAS	Q227K	Lung and thyroid carcinomas, sex cord-stromal tumour (ovary), pituitary adenoma	5
GNAS	Q227E	Adrenal cortical adenoma, pancreatic carcinoma, soft tissue myxoma, adenoma-nodule-goitre (thyroid)	5
GNAS	Q227P	Pancreatic neoplasm	1
GNAQ	Q209L	Uveal , skin and central nervous system malignant melanomas, uveal and meninges melanocytomas, benign melanocytic nevus, soft tissue haemangioma	406
GNAQ	Q209P	Uveal and skin malignant melanomas, central nervous system glioma, germ cell tumour of the testis, meninges melanocytoma, benign melanocytic nevus, soft tissue haemangioma	378
GNAQ	Q209H	Uveal and skin malignant melanomas, benign melanocytic nevus, soft tissue haemangioma	42
GNAQ	Q209R	Uveal and skin malignant melanomas, benign melanocytic nevus, soft tissue haemangioma	18
GNAQ	Q209K	Uveal malignant melanoma	1
GNAQ	Q209Y	Uveal malignant melanoma	1

GNA11	Q209L	Uveal and skin malignant melanomas, meninges melanocytoma, benign melanocytic nevus, soft tissue haemangioma	553
GNA11	Q209H	Uveal and skin malignant melanomas, adrenal cortical adenoma, soft tissue haemangioma	16
GNA11	Q209P	Uveal malignant melanoma, meninges melanocytoma, soft tissue haemangioma, ovarian malignant potential (borderline) tumour	10
GNA11	Q209R	Uveal and skin malignant melanomas	6
GNA11	Q209V	Uveal malignant melanoma	2
GNA11	Q209A	Uveal malignant melanoma	1
GNA11	Q209K	Prostate carcinoma	1
GNA13	Q226E	Skin malignant melanomas	1
